# X-linked recessive TLR7 deficiency in ~1% of men under 60 years old with life-threatening COVID-19

**DOI:** 10.1126/sciimmunol.abl4348

**Published:** 2021-08-19

**Authors:** Takaki Asano, Bertrand Boisson, Fanny Onodi, Daniela Matuozzo, Marcela Moncada-Velez, Majistor Raj Luxman Maglorius Renkilaraj, Peng Zhang, Laurent Meertens, Alexandre Bolze, Marie Materna, Sarantis Korniotis, Adrian Gervais, Estelle Talouarn, Benedetta Bigio, Yoann Seeleuthner, Kaya Bilguvar, Yu Zhang, Anna-Lena Neehus, Masato Ogishi, Simon J. Pelham, Tom Le Voyer, Jérémie Rosain, Quentin Philippot, Pere Soler-Palacín, Roger Colobran, Andrea Martin-Nalda, Jacques G. Rivière, Yacine Tandjaoui-Lambiotte, Khalil Chaïbi, Mohammad Shahrooei, Ilad Alavi Darazam, Nasrin Alipour Olyaei, Davood Mansouri, Nevin Hatipoğlu, Figen Palabiyik, Tayfun Ozcelik, Giuseppe Novelli, Antonio Novelli, Giorgio Casari, Alessandro Aiuti, Paola Carrera, Simone Bondesan, Federica Barzaghi, Patrizia Rovere-Querini, Cristina Tresoldi, Jose Luis Franco, Julian Rojas, Luis Felipe Reyes, Ingrid G. Bustos, Andres Augusto Arias, Guillaume Morelle, Kyheng Christèle, Jesús Troya, Laura Planas-Serra, Agatha Schlüter, Marta Gut, Aurora Pujol, Luis M. Allende, Carlos Rodriguez-Gallego, Carlos Flores, Oscar Cabrera-Marante, Daniel E. Pleguezuelo, Rebeca Pérez de Diego, Sevgi Keles, Gokhan Aytekin, Ozge Metin Akcan, Yenan T. Bryceson, Peter Bergman, Petter Brodin, Daniel Smole, C.I. Edvard Smith, Anna-Carin Norlin, Tessa M. Campbell, Laura E. Covill, Lennart Hammarström, Qiang Pan-Hammarström, Hassan Abolhassani, Shrikant Mane, Nico Marr, Manar Ata, Fatima Al Ali, Taushif Khan, András N. Spaan, Clifton L. Dalgard, Paolo Bonfanti, Andrea Biondi, Sarah Tubiana, Charles Burdet, Robert Nussbaum, Amanda Kahn-Kirby, Andrew L. Snow, COVID Human Genetic Effort, COVID-STORM Clinicians, COVID Clinicians, Imagine COVID Group, French COVID Cohort Study Group, CoV-Contact Cohort, Amsterdam UMC Covid-, Biobank, NIAID-USUHS COVID Study Group, Jacinta Bustamante, Anne Puel, Stéphanie Boisson-Dupuis, Shen-Ying Zhang, Vivien Béziat, Richard P. Lifton, Paul Bastard, Luigi D. Notarangelo, Laurent Abel, Helen C. Su, Emmanuelle Jouanguy, Ali Amara, Vassili Soumelis, Aurélie Cobat, Qian Zhang, Jean-Laurent Casanova, Laurent Abel, Alessandro Aiuti, Saleh Al-Muhsen, Fahd Al-Mulla, Mark S. Anderson, Evangelos Andreakos, Andrés A. Arias, Hagit Baris Feldman, Alexandre Belot, Catherine M. Biggs, Dusan Bogunovic, Alexandre Bolze, Anastasiia Bondarenko, Ahmed A. Bousfiha, Petter Brodin, Yenan Bryceson, Carlos D. Bustamante, Manish J. Butte, Giorgio Casari, Samya Chakravorty, John Christodoulou, Antonio Condino-Neto, Stefan N. Constantinescu, Megan A. Cooper, Clifton L. Dalgard, Murkesh Desai, Beth A. Drolet, Jamila El Baghdadi, Sara Espinosa-Padilla, Jacques Fellay, Carlos Flores, José Luis Franco, Antoine Froidure, Peter K. Gregersen, Filomeen Haerynck, David Hagin, Rabih Halwani, Lennart Hammarström, James R. Heath, Sarah E. Henrickson, Elena W.Y. Hsieh, Eystein Husebye, Kohsuke Imai, Yuval Itan, Erich D. Jarvis, Timokratis Karamitros, Kai Kisand, Cheng-Lung Ku, Yu-Lung Lau, Yun Ling, Carrie L. Lucas, Tom Maniatis, Davood Mansouri, László Maródi, Isabelle Meyts, Joshua D. Milner, Kristina Mironska, Trine H. Mogensen, Tomohiro Morio, Lisa F.P. Ng, Luigi D. Notarangelo, Antonio Novelli, Giuseppe Novelli, Cliona O'Farrelly, Satoshi Okada, Tayfun Ozcelik, Qiang Pan-Hammarström, Rebeca Perez de Diego, Anna M. Planas, Carolina Prando, Aurora Pujol, Lluis Quintana-Murci, Laurent Renia, Igor Resnick, Carlos Rodríguez-Gallego, Vanessa Sancho-Shimizu, Anna Sediva, Mikko R.J. Seppänen, Mohammed Shahrooei, Anna Shcherbina, Ondrej Slaby, Andrew L. Snow, Pere Soler-Palacín, András N. Spaan, Ivan Tancevski, Stuart G. Tangye, Ahmad Abou Tayoun, Sathishkumar Ramaswamy, Stuart E Turvey, K M Furkan Uddin, Mohammed J. Uddin, Diederik van de Beek, Donald C. Vinh, Horst von Bernuth, Mayana Zatz, Pawel Zawadzki, Helen C. Su, Jean-Laurent Casanova, Giuseppe Foti, Giacomo Bellani, Giuseppe Citerio, Ernesto Contro, Alberto Pesci, Maria Grazia Valsecchi, Marina Cazzaniga, Jorge Abad, Giulia Accordino, Cristian Achille, Sergio Aguilera-Albesa, Aina Aguiló-Cucurull, Alessandro Aiuti, Esra Akyüz Özkan, Ilad Alavi Darazam, Jonathan Antonio Roblero Albisures, Juan C Aldave, Miquel Alfonso Ramos, Taj Ali Khan, Anna Aliberti, Seyed Alireza Nadji, Gulsum Alkan, Suzan A. AlKhater, Jerome Allardet-Servent, Luis M Allende, Rebeca Alonso-Arias, Mohammed S Alshahrani, Laia Alsina, Marie-Alexandra Alyanakian, Blanca Amador Borrero, Zahir Amoura, Arnau Antolí, Romain Arrestier, Mélodie Aubart, Teresa Auguet, Iryna Avramenko, Gökhan Aytekin, Axelle Azot, Seiamak Bahram, Fanny Bajolle, Fausto Baldanti, Aurélie Baldolli, Maite Ballester, Hagit Baris Feldman, Benoit Barrou, Federica Barzagh, Sabrina Basso, Gulsum Iclal Bayhan, Alexandre Belot, Liliana Bezrodnik, Agurtzane Bilbao, Geraldine Blanchard-Rohner, Ignacio Blanco, Adeline Blandinières, Daniel Blázquez-Gamero, Alexandre Bleibtreu, Marketa Bloomfield, Mireia Bolivar-Prados, Anastasiia Bondarenko, Alessandro Borghesi, Raphael Borie, Elisabeth Botdhlo-Nevers, Ahmed A Bousfiha, Aurore Bousquet, David Boutolleau, Claire Bouvattier, Oksana Boyarchuk, Juliette Bravais, M. Luisa Briones, Marie-Eve Brunner, Raffaele Bruno, Maria Rita P Bueno, Huda Bukhari, Jacinta Bustamante, Juan José Cáceres Agra, Ruggero Capra, Raphael Carapito, Maria Carrabba, Giorgio Casari, Carlos Casasnovas, Marion Caseris, Irene Cassaniti, Martin Castelle, Francesco Castelli, Martín Castillo de Vera, Mateus V Castro, Emilie Catherinot, Jale Bengi Celik, Alessandro Ceschi, Martin Chalumeau, Bruno Charbit, Matthew P. Cheng, Père Clavé, Bonaventura Clotet, Anna Codina, Yves Cohen, Roger Colobran, Cloé Comarmond, Alain Combes, Patrizia Comoli, Angelo G Corsico, Taner Coşkuner, Aleksandar Cvetkovski, Cyril Cyrus, David Dalmau, François Danion, David Ross Darley, Vincent Das, Nicolas Dauby, Stéphane Dauger, Paul De Munter, Loic de Pontual, Amin Dehban, Geoffroy Delplancq, Alexandre Demoule, Isabelle Desguerre, Antonio Di Sabatino, Jean-Luc Diehl, Stephanie Dobbelaere, Elena Domínguez-Garrido, Clément Dubost, Olov Ekwall, Şefika Elmas Bozdemir, Marwa H Elnagdy, Melike Emiroglu, Akifumi Endo, Emine Hafize Erdeniz, Selma Erol Aytekin, Maria Pilar Etxart Lasa, Romain Euvrard, Giovanna Fabio, Laurence Faivre, Antonin Falck, Muriel Fartoukh, Morgane Faure, Miguel Fernandez Arquero, Ricard Ferrer, Jose Ferreres, Carlos Flores, Bruno Francois, Victoria Fumadó, Kitty S C Fung, Francesca Fusco, Alenka Gagro, Blanca Garcia Solis, Pascale Gaussem, Zeynep Gayretli, Juana Gil-Herrera, Laurent Gilardin, Audrey Giraud Gatineau, Mònica Girona-Alarcón, Karen Alejandra Cifuentes Godínez, Jean-Christophe Goffard, Nacho Gonzales, Luis I Gonzalez-Granado, Rafaela González-Montelongo, Antoine Guerder, Belgin Gülhan, Victor Daniel Gumucio, Leif Gunnar Hanitsch, Jan Gunst, Marta Gut, Jérôme Hadjadj, Filomeen Haerynck, Rabih Halwani, Lennart Hammarström, Selda Hancerli, Tetyana Hariyan, Nevin Hatipoglu, Deniz Heppekcan, Elisa Hernandez-Brito, Po-ki Ho, María Soledad Holanda-Peña, Juan P Horcajada, Sami Hraiech, Linda Humbert, Ivan F N Hung, Alejandro D. Iglesias, Antonio Íñigo-Campos, Matthieu Jamme, María Jesús Arranz, Marie-Thérèse Jimeno, Iolanda Jordan, Saliha Kanık Yüksek, Yalcin Burak Kara, Aydın Karahan, Adem Karbuz, Kadriye Kart Yasar, Ozgur Kasapcopur, Kenichi Kashimada, Sevgi Keles, Yasemin Kendir Demirkol, Yasutoshi Kido, Can Kizil, Ahmet Osman Kılıç, Adam Klocperk, Antonia Koutsoukou, Zbigniew J. Król, Hatem Ksouri, Paul Kuentz, Arthur M C Kwan, Yat Wah M Kwan, Janette S Y Kwok, Jean-Christophe Lagier, David S Y Lam, Vicky Lampropoulou, Fanny Lanternier, Yu-Lung LAU, Fleur Le Bourgeois, Yee-Sin Leo, Rafael Leon Lopez, Daniel Leung, Michael Levin, Michael Levy, Romain Lévy, Zhi Li, Daniele Lilleri, Edson Jose Adrian Bolanos Lima, Agnes Linglart, Eduardo López-Collazo, José M. Lorenzo-Salazar, Céline Louapre, Catherine Lubetzki, Kwok-Cheung Lung, Charles-Edouard Luyt, David C Lye, Cinthia Magnone, Davood Mansouri, Enrico Marchioni, Carola Marioli, Majid Marjani, Laura Marques, Jesus Marquez Pereira, Andrea Martín-Nalda, David Martínez Pueyo, Javier Martinez-Picado, Iciar Marzana, Carmen Mata-Martínez, Alexis Mathian, Larissa RB Matos, Gail V Matthews, Julien Mayaux, Raquel McLaughlin-Garcia, Philippe Meersseman, Jean-Louis Mège, Armand Mekontso-Dessap, Isabelle Melki, Federica Meloni, Jean-François Meritet, Paolo Merlani, Özge Metin Akcan, Isabelle Meyts, Mehdi Mezidi, Isabelle Migeotte, Maude Millereux, Matthieu Million, Tristan Mirault, Clotilde Mircher, Mehdi Mirsaeidi, Yoko Mizoguchi, Bhavi P Modi, Francesco Mojoli, Elsa Moncomble, Abián Montesdeoca Melián, Antonio Morales Martinez, Francisco Morandeira, Pierre-Emmanuel Morange, Cléemence Mordacq, Guillaume Morelle, Stéphane J Mouly, Adrián Muñoz-Barrera, Cyril Nafati, Shintaro Nagashima, Yu Nakagama, Bénédicte Neven, João Farela Neves, Lisa FP Ng, Yuk-Yung Ng, Hubert Nielly, Yeray Novoa Medina, Esmeralda Nuñez Cuadros, J. Gonzalo Ocejo-Vinyals, Keisuke Okamoto, Mehdi Oualha, Amani Ouedrani, Tayfun Özçelik, Aslinur Ozkaya-Parlakay, Michele Pagani, Qiang Pan-Hammarström, Maria Papadaki, Christophe Parizot, Philippe Parola, Tiffany Pascreau, Stéphane Paul, Estela Paz-Artal, Sigifredo Pedraza, Nancy Carolina González Pellecer, Silvia Pellegrini, Rebeca Pérez de Diego, Xosé Luis Pérez-Fernández, Aurélien Philippe, Quentin Philippot, Adrien Picod, Marc Pineton de Chambrun, Antonio Piralla, Laura Planas-Serra, Dominique Ploin, Julien Poissy, Géraldine Poncelet, Garyphallia Poulakou, Marie S Pouletty, Persia Pourshahnazari, Jia Li Qiu-Chen, Paul Quentric, Thomas Rambaud, Didier Raoult, Violette Raoult, Anne-Sophie Rebillat, Claire Redin, Léa Resmini, Pilar Ricart, Jean-Christophe Richard, Raúl Rigo-Bonnin, Nadia Rivet, Jacques G Rivière, Gemma Rocamora-Blanch, Mathieu P Rodero, Carlos Rodrigo, Luis Antonio Rodriguez, Carlos Rodriguez-Gallego, Agustí Rodriguez-Palmero, Carolina Soledad Romero, Anya Rothenbuhler, Damien Roux, Nikoletta Rovina, Flore Rozenberg, Yvon Ruch, Montse Ruiz, Maria Yolanda Ruiz del Prado, Juan Carlos Ruiz-Rodriguez, Joan Sabater-Riera, Kai Saks, Maria Salagianni, Oliver Sanchez, Adrián Sánchez-Montalvá, Silvia Sánchez-Ramón, Laire Schidlowski, Agatha Schluter, Julien Schmidt, Matthieu Schmidt, Catharina Schuetz, Cyril E Schweitzer, Francesco Scolari, Anna Sediva, Luis Seijo, Analia Gisela Seminario, Damien Sene, Piseth Seng, Sevtap Senoglu, Mikko Seppänen, Alex Serra Llovich, Mohammad Shahrooei, Anna Shcherbina, Virginie Siguret, Eleni Siouti, David M Smadja, Nikaia Smith, Ali Sobh, Xavier Solanich, Jordi Solé-Violán, Catherine Soler, Pere Soler-Palacín, Betül Sözeri, Giulia Maria Stella, Yuriy Stepanovskiy, Annabelle Stoclin, Fabio Taccone, Yacine Tandjaoui-Lambiotte, Jean-Luc Taupin, Simon J Tavernier, Loreto Vidaur Tello, Benjamin Terrier, Guillaume Thiery, Christian Thorball, Karolina Thorn, Caroline Thumerelle, Imran Tipu, Martin Tolstrup, Gabriele Tomasoni, Julie Toubiana, Josep Trenado Alvarez, Vasiliki Triantafyllia, Sophie Trouillet-Assant, Jesús Troya, Owen T Y Tsang, Liina Tserel, Eugene Y K Tso, Alessandra Tucci, Şadiye Kübra Tüter Öz, Matilde Valeria Ursini, Takanori Utsumi, Yurdagul Uzunhan, Pierre Vabres, Juan Valencia-Ramos, Ana Maria Van Den Rym, Isabelle Vandernoot, Valentina Velez-Santamaria, Silvia Patricia Zuniga Veliz, Mateus C Vidigal, Sébastien Viel, Cédric Vilain, Marie E Vilaire-Meunier, Judit Villar-García, Audrey Vincent, Guillaume Vogt, Guillaume Voiriot, Alla Volokha, Fanny Vuotto, Els Wauters, Joost Wauters, Alan K L Wu, Tak-Chiu Wu, Aysun Yahşi, Osman Yesilbas, Mehmet Yildiz, Barnaby E Young, Ufuk Yükselmiş, Mayana Zatz, Marco Zecca, Valentina Zuccaro, Van Praet Jens, Bart N. Lambrecht, Van Braeckel Eva, Bosteels Cédric, Hoste Levi, Hoste Eric, Fré Bauters, Jozefien De Clercq, Heijmans Cathérine, Slabbynck Hans, Naesens Leslie, Benoit Florkin, Cécile Boulanger, Dimitri Vanderlinden, Jean-Philippe Annereau, Luis Briseño-Roa, Olivier Gribouval, Anna Pelet, Laurent Abel, Claire Andrejak, François Angoulvant, Delphine Bachelet, Marie Bartoli, Romain Basmaci, Sylvie Behilill, Marine Beluze, Dehbia Benkerrou, Krishna Bhavsar, Lila Bouadma, Sabelline Bouchez, Maude Bouscambert, Minerva Cervantes-Gonzalez, Anissa Chair, Catherine Chirouze, Alexandra Coelho, Camille Couffignal, Sandrine Couffin-Cadiergues, Eric d’Ortenzio, Marie-Pierre Debray, Lauren Deconinck, Dominique Deplanque, Diane Descamps, Mathilde Desvallée, Alpha Diallo, Alphonsine Diouf, Céline Dorival, François Dubos, Xavier Duval, Brigitte Elharrar, Philippine Eloy, Vincent Enouf, Hélène Esperou, Marina Esposito-Farese, Manuel Etienne, Eglantine Ferrand Devouge, Nathalie Gault, Alexandre Gaymard, Jade Ghosn, Tristan Gigante, Morgane Gilg, Jérémie Guedj, Alexandre Hoctin, Isabelle Hoffmann, Ikram Houas, Jean-Sébastien Hulot, Salma Jaafoura, Ouifiya Kafif, Florentia Kaguelidou, Sabrina Kali, Antoine Khalil, Coralie Khan, Cédric Laouénan, Samira Laribi, Minh Le, Quentin Le Hingrat, Soizic Le Mestre, Hervé Le Nagard, François-Xavier Lescure, Sophie Letrou, Yves Levy, Bruno Lina, Guillaume Lingas, Jean Christophe Lucet, Denis Malvy, Marina Mambert, France Mentré, Amina Meziane, Hugo Mouquet, Jimmy Mullaert, Nadège Neant, Duc Nguyen, Marion Noret, Saad Nseir, Aurélie Papadopoulos, Christelle Paul, Nathan Peiffer-Smadja, Thomas Perpoint, Ventzislava Petrov-Sanchez, Gilles Peytavin, Huong Pham, Olivier Picone, Valentine Piquard, Oriane Puéchal, Christian Rabaud, Manuel Rosa-Calatrava, Bénédicte Rossignol, Patrick Rossignol, Carine Roy, Marion Schneider, Richa Su, Coralie Tardivon, Marie-Capucine Tellier, François Téoulé, Olivier Terrier, Jean-François Timsit, Christelle Tual, Sarah Tubiana, Sylvie Van Der Werf, Noémie Vanel, Aurélie Veislinger, Benoit Visseaux, Aurélie Wiedemann, Yazdan Yazdanpanah, Loubna Alavoine, Sylvie Behillil, Charles Burdet, Charlotte Charpentier, Aline Dechanet, Diane Descamps, Xavier Duval, Jean-Luc Ecobichon, Vincent Enouf, Wahiba Frezouls, Nadhira Houhou, Ouifiya Kafif, Jonathan Lehacaut, Sophie Letrou, Bruno Lina, Jean-Christophe Lucet, Pauline Manchon, Mariama Nouroudine, Valentine Piquard, Caroline Quintin, Michael Thy, Sarah Tubiana, Sylvie van der Werf, Valérie Vignali, Benoit Visseaux, Yazdan Yazdanpanah, Abir Chahine, Nawal Waucquier, Maria-Claire Migaud, Dominique Deplanque, Félix Djossou, Mayka Mergeay-Fabre, Aude Lucarelli, Magalie Demar, Léa Bruneau, Patrick Gérardin, Adrien Maillot, Christine Payet, Bruno Laviolle, Fabrice Laine, Christophe Paris, Mireille Desille-Dugast, Julie Fouchard, Denis Malvy, Duc Nguyen, Thierry Pistone, Pauline Perreau, Valérie Gissot, Carole Le Goas, Samatha Montagne, Lucie Richard, Catherine Chirouze, Kévin Bouiller, Maxime Desmarets, Alexandre Meunier, Benjamin Lefévre, Hélène Jeulin, Karine Legrand, Sandra Lomazzi, Bernard Tardy, Amandine Gagneux-Brunon, Frédérique Bertholon, Elisabeth Botelho-Nevers, Christelle Kouakam, Nicolas Leturque, Layidé Roufai, Karine Amat, Sandrine Couffin-Cadiergues, Hélène Espérou, Samia Hendou, Michiel van Agtmael, Anne Geke Algera, Brent Appelman, Frank van Baarle, Diane Bax, Martijn Beudel, Harm Jan Bogaard, Marije Bomers, Peter Bonta, Lieuwe Bos, Michela Botta, Justin de Brabander, Godelieve de Bree, Sanne de Bruin, David T.P. Buis, Marianna Bugiani, Esther Bulle, Osoul Chouchane, Alex Cloherty, Mirjam Dijkstra, Dave A. Dongelmans, Romein W.G. Dujardin, Paul Elbers, Lucas Fleuren, Suzanne Geerlings, Theo Geijtenbeek, Armand Girbes, Bram Goorhuis, Martin P. Grobusch, Florianne Hafkamp, Laura Hagens, Jorg Hamann, Vanessa Harris, Robert Hemke, Sabine M. Hermans, Leo Heunks, Markus Hollmann, Janneke Horn, Joppe W. Hovius, Menno D. de Jong, Rutger Koning, Endry H.T. Lim, Niels van Mourik, Jeaninne Nellen, Esther J. Nossent, Frederique Paulus, Edgar Peters, Dan A.I. Pina-Fuentes, Tom van der Poll, Bennedikt Preckel, Jan M. Prins, Jorinde Raasveld, Tom Reijnders, Maurits C.F.J. de Rotte, Michiel Schinkel, Marcus J. Schultz, Femke A.P. Schrauwen, Alex Schuurmans, Jaap Schuurmans, Kim Sigaloff, Marleen A. Slim, Patrick Smeele, Marry Smit, Cornelis S. Stijnis, Willemke Stilma, Charlotte Teunissen, Patrick Thoral, Anissa M Tsonas, Pieter R. Tuinman, Marc van der Valk, Denise Veelo, Carolien Volleman, Heder de Vries, Lonneke A. Vught, Michèle van Vugt, Dorien Wouters, A. H (Koos) Zwinderman, Matthijs C. Brouwer, W. Joost Wiersinga, Alexander P.J. Vlaar, Diederik van de Beek, Miranda F. Tompkins, Camille Alba, Andrew L. Snow, Daniel N. Hupalo, John Rosenberger, Gauthaman Sukumar, Matthew D. Wilkerson, Xijun Zhang, Justin Lack, Andrew J. Oler, Kerry Dobbs, Ottavia M. Delmonte, Jeffrey J. Danielson, Andrea Biondi, Laura Rachele Bettini, Mariella D’Angio, Ilaria Beretta, Luisa Imberti, Alessandra Sottini, Virginia Quaresima, Eugenia Quiros-Roldan, Camillo Rossi

**Affiliations:** 1 St. Giles Laboratory of Human Genetics of Infectious Diseases, Rockefeller Branch, The Rockefeller University, New York, NY, USA.; 2 Laboratory of Human Genetics of Infectious Diseases, Necker Branch, INSERM U1163, Necker Hospital for Sick Children, Paris, France.; 3 University of Paris, Imagine Institute, Paris, France.; 4 Laboratory of Genomes & Cell Biology of Disease, INSERM U944, CNRS UMR7212, University of Paris, Research Institute of Saint-Louis, Saint-Louis Hospital, Paris, France.; 5 Helix, San Mateo, CA, USA; 6 University of Paris, INSERM U976, F-75006 Paris, France; 7 Yale Center for Genome Analysis and Department of Genetics, Yale School of Medicine, New Haven, CT, USA.; 8 Laboratory of Clinical Immunology and Microbiology, Division of Intramural Research, NIAID, NIH, Bethesda, MD, USA.; 9 NIAID Clinical Genomics Program, NIH, Laboratory of Clinical Immunology and Microbiology, Division of Intramural Research, NIAID, NIH, Bethesda, MD, USA.; 10 Infection in Immunocompromised Pediatric Patients Research Group, Vall d’Hebron Research Institute (VHIR), Vall d’Hebron University Hospital (HUVH), Vall d’Hebron Barcelona Hospital Campus, Barcelona, Catalonia Spain.; 11 Pediatric Infectious Diseases and Immunodeficiencies Unit, Vall d’Hebron University Hospital (HUVH), Vall d’Hebron Research Institute (VHIR), Vall d’Hebron Barcelona Hospital Campus, Autonomous University of Barcelona (UAB), Barcelona, Catalonia, Spain.; 12 Jeffrey Modell Diagnostic and Research Center for Primary Immunodeficiencies, Barcelona, Catalonia, Spain.; 13 Diagnostic Immunology Group, Vall d’Hebron Research Institute (VHIR), Vall d’Hebron University Hospital (HUVH), Vall d’Hebron Barcelona Hospital Campus, Barcelona, Catalonia, Spain.; 14 Immunology Division, Genetics Department, Vall d’Hebron University Hospital (HUVH), Vall d’Hebron Barcelona Hospital Campus, Autonomous University of Barcelona (UAB), Barcelona, Catalonia, Spain.; 15 AP-HP, Avicenne Hospital, Intensive Care Unit, Bobigny, France.; 16 INSERM U1272 Hypoxia & Lung, Bobigny, France.; 17 Anesthesiology and Critical Care Medicine Department, APHP, Avicenne Hospital, Bobigny, France.; 18 Common and Rare Kidney Diseases, Sorbonne University, INSERM UMR-S 1155, Paris, France.; 19 Specialized Immunology Laboratory of Dr. Shahrooei, Sina Medical Complex, Ahvaz, Iran.; 20 Department of Microbiology and Immunology, Clinical and Diagnostic Immunology, KU Leuven, Leuven, Belgium.; 21 Infectious Diseases and Tropical Medicine Research Center, Shahid Beheshti University of Medical Sciences, Tehran, Iran.; 22 Department of Infectious Diseases and Tropical Medicine, Loghman Hakim Hospital, Shahid Beheshti University of Medical Sciences, Tehran, Iran.; 23 Department of Clinical Immunology and Infectious Diseases, National Research Institute of Tuberculosis and Lung Diseases, Shahid Beheshti University of Medical Sciences, Tehran, Iran.; 24 The Clinical Tuberculosis and Epidemiology Research Center, National Research Institute of Tuberculosis and Lung Diseases (NRITLD), Masih Daneshvari Hospital, Shahid Beheshti, University of Medical Sciences, Tehran, Iran.; 25 Pediatric Respiratory Diseases Research Center, National Research Institute of Tuberculosis and Lung Diseases, Shahid Beheshti, Iran.; 26 Pediatric Infectious Diseases Unit, Bakirkoy Dr. Sadi Konuk Training and Research Hospital, University of Health Sciences, Istanbul, Turkey.; 27 Department of Molecular Biology and Genetics, University of Bilkent, Bilkent-Ankara, Turkey.; 28 Department of Biomedicine and Prevention, University of Rome “Tor Vergata,” Rome, and Neuromed Institute, IRCCS, Pozzilli (IS), Italy.; 29 Laboratory of Medical Genetics, Translational Cytogenomics Research Unit, Bambino Gesù Children Hospital, IRCCS, Rome, Italy.; 30 Vita-Salute San Raffaele University, Milan, Italy.; 31 Clinical Genomics, IRCCS San Raffaele Scientific Institute, Milan, Italy.; 32 San Raffaele Telethon Institute for Gene Therapy (SR-Tiget) and Pediatric Immunohematology Unit and BMT Program, IRCCS San Raffaele Scientific Institute, Milan, Italy.; 33 Division of Immunology, Transplantation and Infectious Diseases, IRCCS San Raffaele Scientific Institute, Milan, Italy.; 34 Molecular Hematology Unit, IRCCS Ospedale San Raffaele, Milan, Italy.; 35 Primary Immunodeficiencies Group, Department of Microbiology and Parasitology, School of Medicine, University of Antioquia, Medellín, Colombia.; 36 Universidad de La Sabana, Chia, Colombia.; 37 School of Microbiology, University of Antioquia UdeA, Medellín, Colombia; 38 Department of General Pediatrics, Hôpital Bicêtre, AP-HP, University of Paris Saclay, Le Kremlin-Bicêtre, France.; 39 Department of Internal Medicine, Infanta Leonor University Hospital, Madrid, Spain.; 40 Neurometabolic Diseases Laboratory, Bellvitge Biomedical Research Institute (IDIBELL), Barcelona, Spain.; 41 Center for Biomedical Research on Rare Diseases (CIBERER), ISCIII, Spain.; 42 CNAG-CRG, Centre for Genomic Regulation (CRG), The Barcelona Institute of Science and Technology (BIST), Baldiri Reixac 4, 08028, Barcelona, Spain.; 43 Catalan Institution of Research and Advanced Studies (ICREA), Barcelona, Spain.; 44 Immunology Department, University Hospital 12 de Octubre, Research Institute Hospital 12 de Octubre (I+12), Madrid, Spain.; 45 Complutense University, Madrid, Spain.; 46 Department of Immunology, University Hospital of Gran Canaria Dr. Negrín, Canarian Health System, Las Palmas de Gran Canaria, Spain.; 47 Department of Clinical Sciences, University of Fernando Pessoa Canarias, Las Palmas de Gran Canaria, Spain.; 48 Genomics Division, Institute of Technology and Renewable Energies (ITER), Santa Cruz de Tenerife, Spain.; 49 CIBER de Enfermedades Respiratorias, Health Institute of Carlos III, Madrid, Spain; 50 Research Unit, University Hospital of N.S. de Candelaria, Santa Cruz de Tenerife, Spain.; 51 Institute of Biomedical technologies (ITB), University of La Laguna, San Cristóbal de La Laguna, Spain.; 52 Institute of Biomedical Research of IdiPAZ, University Hospital “La Paz”, Madrid, Spain.; 53 Necmettin Erbakan University, Meram Medical Faculty, Division of Pediatric Allergy and Immunology, Konya, Turkey.; 54 Konya City Hospital, Division of Allergy and Immunology, Konya, Turkey.; 55 Centre for Hematology and Regenerative Medicine, Department of Medicine, Karolinska Institute, Stockholm, Sweden; 56 Department of Laboratory Medicine, Division of Clinical Microbiology, Karolinska Institute, Stockholm, Sweden; 57 Science for Life Laboratory, Department of Women's and Children's Health, Karolinska Institute, Solna, Sweden; 58 Central Hospital-Anesthesia and Intensive Care Unit, Karlstad, Sweden; 59 Department of Laboratory Medicine, Division of Biomolecular and Cellular Medicine, Karolinska Institute, Stockholm, Sweden; 60 The Immunodeficiency Unit, Infectious Disease Clinic, Karolinska University Hospital, Stockholm, Sweden; 61 Department of Biosciences and Nutrition, Karolinska Institute, Stockholm, Sweden; 62 Research Center for Immunodeficiencies, Pediatrics Center of Excellence, Children's Medical Center, Tehran University of Medical Sciences, Tehran, Iran.; 63 Department of Genetics, Yale University School of Medicine, New Haven, Connecticut, USA.; 64 Department of Immunology, Research Branch, Sidra Medicine, Doha, Qatar.; 65 Department of Medical Microbiology, University Medical Center Utrecht, Utrecht, Netherlands.; 66 Department of Anatomy, Physiology & Genetics Uniformed Services University of the Health Sciences, Bethesda, MD, USA.; 67 The American Genome Center, Uniformed Services University of the Health Sciences, Bethesda, MD, USA.; 68 Department of Infectious Diseases, San Gerardo Hospital–University of Milano-Bicocca, Monza, Italy.; 69 Pediatric Department and Centro Tettamanti-European Reference Network PaedCan, EuroBloodNet, MetabERN-University of Milano-Bicocca-Fondazione MBBM- Ospedale San Gerardo, Monza, Italy.; 70 Centre d'Investigation Clinique, INSERM CIC 1425, Paris, France.; 71 Hôpital Bichat Claude Bernard, APHP, Paris, France.; 72 Université de Paris, IAME, INSERM UMR 1137, Paris, France.; 73 Invitae, San Francisco, CA, USA.; 74 Department of Pharmacology & Molecular Therapeutics, Uniformed Services University of the Health Sciences, Bethesda, MD, USA.; 75 Center for the Study of Primary Immunodeficiencies, Necker Hospital for Sick Children, AP-HP, Paris, France, EU; 76 Laboratory of Genetics and Genomics, The Rockefeller University, New York, NY, USA.; 77 Department of Pathology and Laboratory Medicine, Perelman School of Medicine, University of Pennsylvania, Philadelphia, PA, USA.; 78 APHP, Hôpital Saint-Louis, Department of Immunology-Histocompatibility, 75010 Paris, France.; 79 Howard Hughes Medical Institute, New York, NY, USA.

## Abstract

Autosomal inborn errors of type I IFN immunity and autoantibodies against these cytokines underlie at least 10% of critical COVID-19 pneumonia cases. We report very rare, biochemically deleterious X-linked *TLR7* variants in 16 unrelated male individuals aged 7 to 71 years (mean: 36.7 years) from a cohort of 1,202 male patients aged 0.5 to 99 years (mean: 52.9 years) with unexplained critical COVID-19 pneumonia. None of the 331 asymptomatically or mildly infected male individuals aged 1.3 to 102 years (mean: 38.7 years) tested carry such *TLR7* variants (*p* = 3.5 × 10^−5^). The phenotypes of five hemizygous relatives of index cases infected with SARS-CoV-2 include asymptomatic or mild infection (*n*=2, 5 and 38 years), or moderate (*n*=1, 5 years), severe (*n*=1, 27 years), or critical (*n*=1, 29 years) pneumonia. Two boys (aged 7 and 12 years) from a cohort of 262 male patients with severe COVID-19 pneumonia (mean: 51.0 years) are hemizygous for a deleterious TLR7 variant. The cumulative allele frequency for deleterious *TLR7* variants in the male general population is < 6.5x10^−4^. We also show that blood B cell lines and myeloid cell subsets from the patients do not respond to TLR7 stimulation, a phenotype rescued by wild-type *TLR7*. The patients’ blood plasmacytoid dendritic cells (pDCs) produce low levels of type I IFNs in response to SARS-CoV-2. Overall, X-linked recessive TLR7 deficiency is a highly penetrant genetic etiology of critical COVID-19 pneumonia, in about 1.8% of male patients below the age of 60 years. Human TLR7 and pDCs are essential for protective type I IFN immunity against SARS-CoV-2 in the respiratory tract.

## Teaser

TLR7 and plasmacytoid dendritic cells are essential for type I IFN-dependent immunity to SARS-CoV-2 in the lungs.

## INTRODUCTION

Interindividual clinical variability in the course of SARS-CoV-2 infection is vast, ranging from silent infection to lethal disease (*1*). The greatest risk factor for life-threatening COVID-19 pneumonia is age, with a doubling in risk every five years from the age of five years onward, and a sharp rise after the age of 65 years (*2*, *3*). Other epidemiological risk factors, including common genetic variants, have only modest effects, with odds ratios (ORs) < 2 and typically < 1.5 (*2*). One intriguing observation is the approximately 1.5 times higher risk in men, which seems to be age-independent (*2*–*4*). The COVID Human Genetic Effort consortium (www.covidhge.com) has enrolled an international cohort of patients, with the aim of investigating genetic and immunological causes of life-threatening COVID-19 pneumonia. We previously tested the hypothesis that critical influenza and critical COVID-19 can be allelic (*5*–*7*), and showed that life-threatening COVID-19 pneumonia can be caused by rare inborn errors of autosomal genes controlling TLR3- and IRF7-dependent type I interferon (IFN) immunity (*8*). These disorders were found in 23 men and women aged 17 to 77 years (mean: 48 years). Remarkably, four unrelated patients aged 25 to 50 years had autosomal recessive IFNAR1 (*n*=2) or IRF7 (*n*=2) deficiency. These patients had no previous history of severe viral illness, including influenza pneumonia, implying that these genetic disorders unexpectedly show incomplete penetrance for critical influenza. These findings revealed that TLR3- and IRF7-dependent type I IFN immunity is essential for host defense against SARS-CoV-2 infection in the respiratory tract.

We also found pre-existing neutralizing auto-Abs against type I IFN in at least 10% of the patients from this cohort (*9*). These auto-Abs were found in 101 patients, mostly men (95%), and older members of the cohort, which included patients with inborn errors, as they were aged 25 to 87 years (mean: 65 years). These findings have been replicated in five other cohorts (*10*–*15*). These auto-Abs predated SARS-CoV-2 infection and were highly likely to be causal for critical COVID-19 pneumonia, because (i) they were found in samples drawn before infection in some patients (*9*), (ii) they were found in about 0.3% of the general population before the age of 65 years (*9*), (iii) they were absent from patients with asymptomatic or paucisymptomatic (mild) SARS-CoV-2 infection (*9*), (iv) they were of childhood onset in patients with various disorders — including autoimmune polyendocrinopathy type I (APS-1) — known to be at very high risk of life-threatening COVID-19 (*16*), and (v) they have been shown to underlie a third of adverse reactions to the live attenuated viral vaccine for yellow fever (*17*). Collectively, these studies showed that type I IFNs are essential for protective immunity to SARS-CoV-2 in the respiratory tract, but are otherwise surprisingly redundant. Auto-Abs against type I IFNs also provide a first explanation for both the biased sex ratio and the higher risk of critical COVID-19 in patients over the age of 65 years. Here, we tested the hypothesis that critical and unexplained COVID-19 pneumonia in men may be due to rare variants on the X- chromosome.

## RESULTS

### Enrichment for very rare *TLR7* non-synonymous variants in male patients

**Fig. 1:**
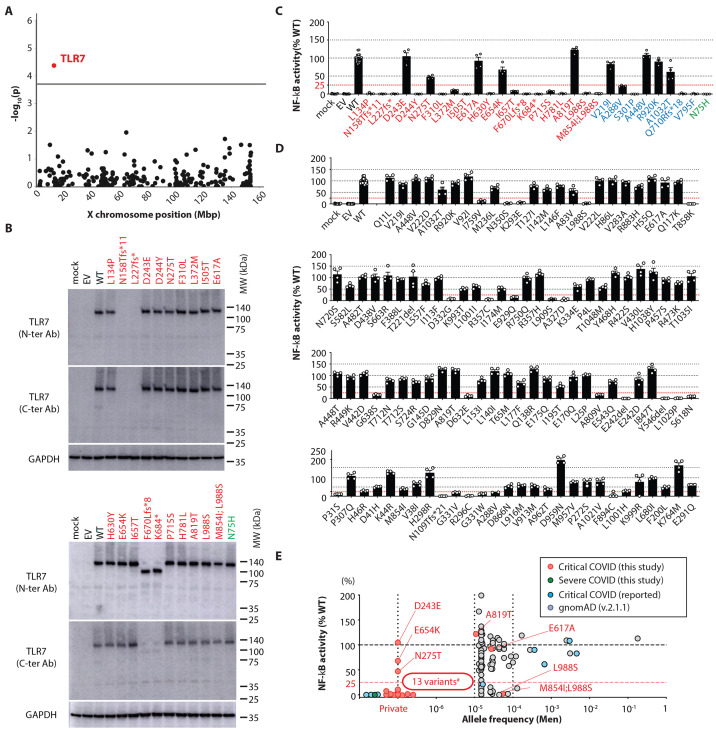
**Enrichment in rare *TLR7* deleterious alleles among men with critical COVID-19 pneumonia. (A)** Manhattan plot showing the results of the variant enrichment test for the 190 genes of the X chromosome with at least 5 patients carrying non-synonymous variants. The gray line indicates the corresponding Bonferroni-corrected significance threshold. (**B**) Western blot of extracts from non-transfected HEK293T cells (mock), HEK293T cells transfected with pCMV6 empty vector (EV), the wild-type (WT) *TLR7* allele, or one of the *TLR7* variant alleles of interest. All extracts were probed with monoclonal antibodies specific for the leucine-rich repeats to the N terminus (N-ter) or amino-acid 1,000 to the C terminus (C-ter) within the human TLR7 protein. **(C) (D)** Luciferase assay on HEK293T cells transfected with the pGL4.32 luciferase reporter construct and an expression vector for *Renilla* luciferase together with no vector (mock), EV, WT, or *TLR7* variants: (C) 21 variants found in our cohort and eight previously reported variants, (D) 109 variants found in male individuals from the gnomAD database. After 24 hours, transfected cells were left untreated or were treated by incubation with 1 μg/mL R848 for 24 hours. These data were established from two independent experiments. The *y*-axis represents NF-κB transcriptional activity as a percentage of the WT. The *x*-axis indicates the alleles used for transfection. **(E)** Diagram showing the correlation between allele frequency and NF-κB activity (% of WT). The 20 variants from 21 patients with critical SARS-CoV-2 from our cohort are shown in red, one variant from 2 patients with severe SARS-CoV-2 from our cohort are shown in green, the eight previously reported variants are shown in blue and the 109 variants found in the general population (allele frequency above 10^−5^ in men) are shown in gray. Activity of all LOF/hypomorphic alleles compared to WT allele were statistically significance (one-way ANOVA with Dunnett’s post hoc test, P < 0.01).

We tested the hypothesis of genetic homogeneity for X-linked recessive disorders in male individuals with critical COVID-19 pneumonia (hereafter referred to as “patients”, see Materials and Methods). We analyzed an international cohort of 1,202 unrelated male patients aged 6 months to 99 years (mean: 52.9 years) that possessed no known inborn errors of TLR3- and IRF7-dependent type I IFN immunity (*8*) and without neutralizing auto-Abs against type I IFNs (*9*) (reported in an accompanying paper (*79*)) (Table S1). We also analyzed 331 asymptomatic or paucisymptomatic infected male subjects aged 1.3 to 102 years (mean: 38.7 years), with positive results for PCR and/or serological screening for SARS-CoV-2 infection (hereafter referred to as “controls”) (Table S1). We sequenced the exomes (*n*=1,035) or genomes (*n*=498) of these patients and controls. We selected in-frame and out-of-frame non-synonymous variants of protein-coding exons that are very rare, that is, with a minor allele frequency (MAF) below 10^−4^ in the full gnomAD database (v2.1.1) containing sequences from both male and female individuals. We compared the proportions of patients and controls carrying at least one qualifying variant, by Firth bias-corrected logistic regression adjusted for age and ethnicity (*18*) (Fig. S1A). We found non-synonymous variants in at least five patients for 226 of 731 genes on the X chromosome, resulting in a Bonferroni-corrected significance threshold of 2.2x10^−4^ (Data file S1). *TLR7* was the highest ranked of these genes (uncorrected *P*-value = 3.5×10^−5^) and the only gene that remained significant after correction for multiple testing (corrected *P*-value =7.8×10^−3^), with 21 unrelated patients carrying one very rare (*n*=4 patients), two very rare (*n*=1 patient), or one private (*n*=16 patients) non-synonymous variant (Fig. 1A, Table S2). One variant (L988S) was recurrent, found in three patients, including a patient carrying two very rare variants (M854I;L988S). No such variants were found in the controls. The same analysis performed on very rare (MAF<10^−4^) synonymous *TLR7* variants showed no enrichment in patients (one carrier) relative to controls (three carriers).

Human TLR7 is an endosomal receptor of ribonucleic acids expressed by B cells and myeloid subsets (*19*–*23*), the stimulation of which in plasmacytoid dendritic cells (pDCs) results in the production of large amounts of type I IFN (*24*–*26*). We observed no significant enrichment for coding non-synonymous variants of the X-linked gene *TLR8* (*P*-value = 0.68, Table S2), the product of which, TLR8, is endosomal and can be stimulated by some synthetic TLR7 agonists, with an expression pattern and signaling pathway overlapping those of TLR7 (*27*, *28*). Unlike TLR7, TLR8 is expressed on granulocytes but not on pDCs, possibly accounting for its gain-of-function mutations underlying a phenotype different from type I interferonopathies (*29*–*31*). Overall, we found an enrichment in very rare or private non-synonymous *TLR7* variants among the male patients with critical COVID-19 pneumonia (*n*=21, 1.7%) of our cohort (*n*=1,202), including one man over the age of 60 years.

### The *TLR7* mutant alleles of 16 of the 21 unrelated patients with critical COVID-19 pneumonia are biochemically deleterious

The 21 unrelated patients carried 20 different *TLR7* alleles. We expressed the 20 TLR7 mutant proteins in human embryonic kidney (HEK) 293T cells, which have no endogenous TLR7 and TLR8 expression (*32*), by transient transfection with the corresponding cDNAs. Immunoblotting of protein extracts with a TLR7-specific mAb showed an absence of TLR7 protein for p.N158Tfs*11 and p.L227fs* and the presence of truncated proteins for K684* and F670Lfs*8 (Fig. 1B). The other mutant TLR7 proteins were produced in normal amounts (Fig. 1B). We tested their function by cotransfection with an NF-κB-specific luciferase reporter. We measured luciferase activity upon stimulation with R848, an agonist of both TLR7 and TLR8 (Fig. 1C). Twelve of the 20 alleles were loss-of-function (LOF) (including L988S in two patients, and M854I;L988S in another), three (p.L372M, p.I657T and p.P715S) were hypomorphic (activity < 25%), and the remaining five were neutral (Fig. 1C, Data file S2). Similar results were obtained with imiquimod and CL264, two TLR7-specific agonists (Fig. S1B, S1C). We also tested eight other private (p.S301P, p.Q710Rfs*18, p.V795F), very rare (MAF <10^−4^; p.A288V) or rare (MAF between 10^−4^ and 10^−2^; p.V219I, p.A448V, p.R920K, p.A1032T) *TLR7* variants previously reported in patients with critical COVID-19 (*33*, *34*). These variants were expressed as truncated or full-length proteins (Fig. S1D). The proteins encoded by the three private variants were found to be LOF, that encoded by the very rare variant (p.A288V) was hypomorphic, and those encoded by the four rare variants were neutral (Fig. 1C, Fig. S1B). Collectively, these findings suggest that 16 of the 21 patients in our cohort (Table 1), as well as only 6 of the previously reported 12 patients carry deleterious *TLR7* variants.

**Table 1:** X-linked *TLR7* deleterious variants in 16 unrelated male patients with life-threatening COVID-19 pneumonia.

**Patient**	**Genotype**	**Age [years]**	**Ethnicity**	**Ancestry/residence**	**Outcome**
P1	L134P/Y	45	Admixed American	Paraguay/Spain	Survived
P2	N158Tfs11*/Y	60	European	France	Deceased
P3	L227fs*/Y	34	Middle East	Iran	Survived
P4	D244Y/Y	13	Middle East	Turkey	Survived
P5	F310L/Y	39	Middle East	Iran	Survived
P6	L372M	7	Caucasian (Central Asia based on GME Variome)	Iran	Survived
P7	I505T/Y	55	European	Italy	Survived
P8	H630Y/Y	50	European	Spain	Survived
P9	I657T/Y	18	European	Italy	Survived
P10	F670Lfs*8	31	European	Sweden	Survived
P11*	F670Lfs*8	29	European	Sweden	Survived
P12	K684*/Y	30	European	Spain	Survived
P13	P715S/Y	40	Latino	Colombia	Survived
P14	H781L/Y	13	Middle East	Russia/France	Survived
P15	L988S/Y	26	Middle East	Iran	Deceased
P16	L988S/Y	20	Middle East	Turkey	Survived
P17	M854I;L988S/Y	71	European	Italy	Survived

* P10’s brother (not included in the cohort of 1,202 critical patients with critical COVID-19 pneumonia).

GME Variome, Greater Middle Eastern Variome Project

### The cumulative MAF of deleterious TLR7 alleles is < 6.5x10^−4^

We also investigated the production and function of all 100 remaining non-synonymous *TLR7* variants identified in the general population (141,456 individuals in gnomAD v2.1) that had been reported in men or had a general MAF > 10^−5^ (Fig. 1D and Fig. S1E, Data file S2). In total, 96 of these variants were missense and three were in-frame small deletions; 10 were weakly expressed, whereas the others had normal levels of expression (Fig. S1F, Data file S2). One variant was a small deletion creating a frameshift found in one man and resulting in an absence of protein production (Fig. S1F, Data file S2). Seven of the 100 variants were LOF and 15 were hypomorphic (< 25% activity) (Data file S2). There were, thus, 24 deleterious *TLR7* variants, including the L988S and A288V variants found in four patients with critical COVID-19 pneumonia. Each of these 24 deleterious variants had an individual MAF < 1.3x10^−4^ in men and their cumulative MAF in men was 6.5 x10^−4^ (Data file S2, Table S3). The cumulative MAF of strictly LOF *TLR7* alleles (excluding hypomorphic alleles) in men is about 2.2 x10^−4^ (Data file S2). Overall, we found 12 LOF and three hypomorphic *TLR7* alleles in 16 unrelated men with critical COVID-19 pneumonia, whereas deleterious alleles were not found in men with asymptomatic or paucisymptomatic infection. Moreover, deleterious *TLR7* alleles in the general population had individual and cumulative MAF values in men of < 1.3x10^−4^ and < 6.5x10^−4^, respectively (Fig. 1E, Data file S2). The rarity of TLR7 deficiency in the general population is consistent with TLR7 deficiency underlying critical COVID-19. Collectively, these findings suggest that X-linked recessive (XR) TLR7 deficiency is a genetic etiology of life-threatening COVID-19 pneumonia in men.

### High clinical penetrance of inherited TLR7 deficiency in the patients’ families

**Fig. 2:**
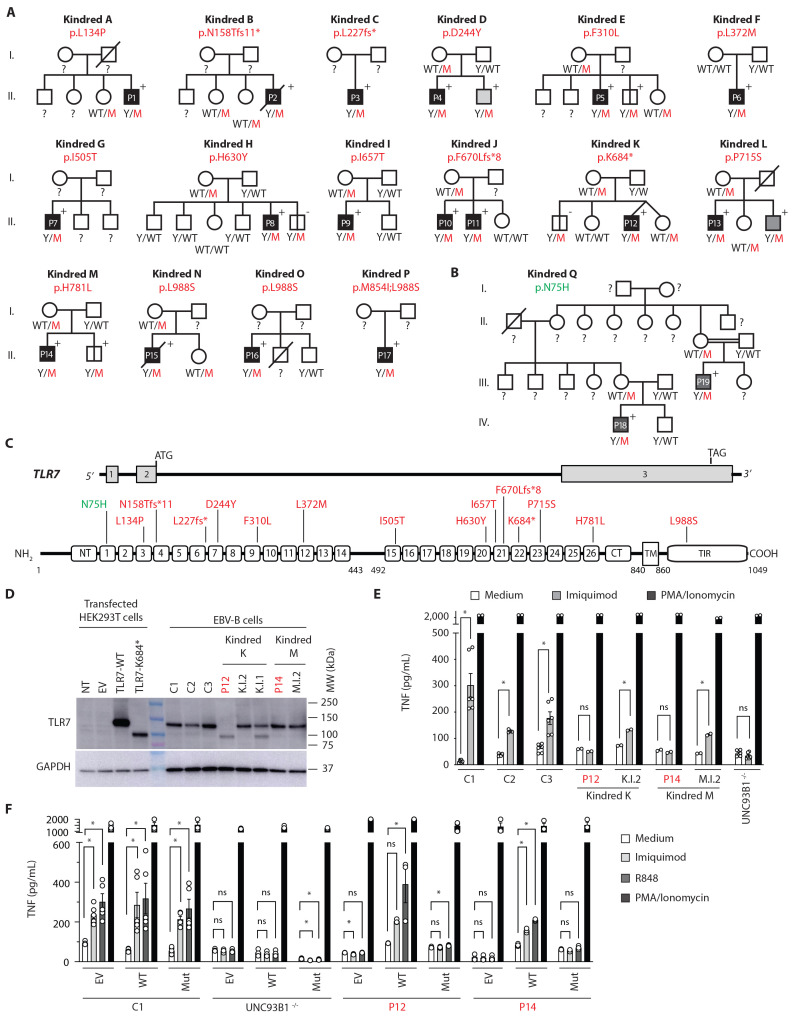
**X-linked recessive TLR7 deficiency in 16 kindreds. (A)** Pedigrees of the 16 kindreds containing 17 patients with life-threatening COVID-19 pneumonia (P1-17) bearing deleterious *TLR7* alleles. The mutations are indicated above each pedigree. Solid black symbols indicate patients with critical COVID-19, and solid dark gray symbols indicate severe cases and solid light gray symbols indicate mild/moderate cases. The genotype is indicated under each symbol, with M corresponding to the mutation found in each kindred. ‘+’ and ‘-’ indicate the presence and absence, respectively, of antibodies against SARS-CoV-2 in the serum of the individual. Asymptomatic or paucisymptomatic family members hemizygous for the mutation are indicated by bold vertical lines. (**B**) Pedigree of one kindred containing two patients with severe COVID-19. (**C**) Schematic representation of TLR7. The upper part represents the genomic organization of the *TLR7* locus, with rectangles for the various exons of the gene, and exon numbers indicated within the rectangle. The bottom part shows the primary structure of TLR7. The N-terminal portion and the leucine-rich repeat containing 26 leucine residues are located in the lumen of the endosome, and TM indicates the transmembrane domain. The Toll/interleukin-1 (IL-1) receptor (TIR) domain is cytoplasmic. The deleterious mutations reported in this study are indicated. (**D**) TLR7 expression in unstimulated EBV-B cells from two patients with XR TLR7 deficiency (P12 and P14), the fathers of P12 and P14, and the mother of P12, and three healthy donors (Control 1 to 3), determined by Western blotting with detection with a specific TLR7 antibody. (**E**) TNF production by XR TLR7-deficient EBV-B cells from two independent experiments. Cells were either left untreated or were stimulated with 5 μg/mL imiquimod (gray), or 25 ng/mL PMA and 0.25 μM ionomycin (black) for 24 hours and TNF production were measured by ELISA. (**F**) TNF production in XR TLR7-deficient EBV-B cells re-expressing WT TLR7 from three independent experiments. EBV-B cells from a control, P12, P14, or an UNC-93B-deficient patient, cultured in the presence of IRAK4 inhibitor (PF06650833- 5 μM) were transduced with lentiviral particles that were empty or contained the WT TLR7 or mutant TLR7 cDNA. The cells were incubated for 24 hours without IRAK4 inhibitor and were then left untreated or were stimulated with 5 μg/mL imiquimod (light gray), 1 μg/mL R848 (dark gray), or 25 ng/mL PMA and 0.25 μM ionomycin (black) for 24 hours, and TNF production were measured by ELISA. Statistical tests were performed using one-way ANOVA with Dunnett’s post hoc test (*: P < 0.05, ns: not significant).

The 16 patients were of three major ethnic origins, as confirmed by principal component analysis (PCA) of their exomes or genomes (*35*), and they were resident in seven countries (France *n*=2, Spain *n*=3, Italy *n*=3, Turkey *n*=2, Sweden *n*=1, Iran *n*=4, Colombia *n*=1) (Fig. 2A, Fig. 2C, Fig. S1, Table 1, Data file S3). The patients were hospitalized for critical COVID-19 between March 2020 and June 2021. Blood samples (diluted 1/10) from these 16 patients contained no auto-Abs neutralizing 10 ng/mL IFN-α2 and/or -ω (*9*) (*79*). The patients were aged 7 to 71 years and their mean age was lower than that of the total cohort (mean age of 34.4 years, versus 52.9 years for the total cohort, in which age ranged from 0.5 to 99 years). TLR7-deficient patients accounted for about 1.8% of the patients below the age of 60 years (15 patients) and 1.3% of the entire cohort (16 patients). Two patients died and 14 survived (Fig. 2A, Table 1). Sanger sequencing of the *TLR7* locus in the relatives of these patients identified the deleterious alleles in 16 heterozygous women from eleven families and seven hemizygous men from seven families (Fig. 2A). Based on the ten DNA samples available from the patients’ mothers, only one of the *TLR7* variants (L372M) was de novo in the index case. Five of the seven hemizygous relatives of the index cases had antibodies against SARS-CoV-2 (Fig. 2A, Data file S3). One 29-year-old adult (Kindred J, P11) was hospitalized for critical pneumonia, and another 27-year-old adult (L.II.3) was hospitalized for severe pneumonia (with low-flow oxygen (<6L/min)). The remaining three were two five-year-old boys, one of whom had been hospitalized for moderate COVID-19 pneumonia (without oxygen therapy) (D.II.2), the other having no relevant clinical history (M.II.2), and one 38-year-old adult with no relevant clinical history (E.II.4) (Data file S3). The other two male carriers did not report SARS-CoV-2 infection and had negative serological results for antibodies against the SARS-CoV-2 S and N proteins.

### Inherited TLR7 deficiency in patients with severe COVID-19 pneumonia

Given these results, we also analyzed 262 other, unrelated male patients with severe (but not critical) COVID-19 pneumonia (mean age: 51.0 years). We identified a new private LOF variant (p.N75H) in two male patients from two Turkish families (P18 and P19), aged 12 and 7 years, respectively, who were subsequently found to be fourth-degree relatives (Fig. 1B, 1C, 1D, Fig. 2B, Fig. S1B, Data file S2, Data file S3). Their mothers are heterozygous for this variant. The clinical penetrance of critical COVID-19 in men is therefore high, but not complete, and TLR7 deficiency can also underlie severe COVID-19. The absence of biochemically deleterious *TLR8* variants in our cohort of patients with critical COVID-19 (Fig. S2) and its lack of expression on pDCs suggest that *TLR8* is not a modifier of the SARS-CoV-2-related clinical phenotype of TLR7 deficiency, although it is adjacent to *TLR7* on the X chromosome and can be stimulated by overlapping molecules. Perhaps more relevant to the understanding of the incomplete penetrance is the age of the patients. Of the 23 male patients carrying deleterious alleles of *TLR7* infected with SARS-CoV-2, the 20 patients who developed severe (*n*=3) or critical (*n*=17) COVID-19 were aged 7-71 years (mean: 32.4 years) whereas the three patients who developed asymptomatic, mild, or moderate infection were younger: 5, 5, and 38 years (mean: 16 years). Blood pDC counts decrease with age (*36*–*38*), and this may contribute to the apparent increase in penetrance with age. In addition, a VirScan study of the serum samples of five index cases and three TLR7 hemizygous relatives revealed prior infection with diverse viruses (Fig. S3). None had previously been hospitalized for a severe viral illness, including influenza pneumonia. This cohort of patients thus suggests that TLR7 deficiency does not underlie severe disease caused by common viral infections other than SARS-CoV-2, or if so, with lower penetrance.

### Deleterious *TLR7* alleles abolish B cell responses to TLR7 agonists

As a first approach to testing the impact of deleterious *TLR7* alleles in the patients’ cells, we tested Epstein-Barr virus-transformed B cell lines (EBV-B cells) from healthy controls and patients carrying the hemizygous p.K684* (P12) or p.H781L (P14) variants. The endogenous expression of the p.H781L TLR7 protein was normal, whereas p.K684* generated a truncated protein (Fig. 2D). In response to agonists of TLR7 (imiquimod) or TLR7 plus TLR8 (R848), the EBV-B cell lines carrying these two mutations failed to produce TNF (Fig. 2E, Fig. S4A, S4B). The lentiviral transduction of these TLR7-deficient EBV-B cells (from P12 and P14) with a WT TLR7 cDNA was unsuccessful, despite numerous attempts, and this was also the case for control EBV-B cells, perhaps because the overproduction of TLR7 is toxic in B cells (*39*). Consistent with this view, we were able to express this cDNA in IRAK4- or MyD88-deficient EBV-B cells. We therefore investigated whether the addition of an IRAK4 inhibitor (PF06650833) would permit the expression of WT TLR7 in control and TLR7-mutated EBV-B cells. This approach was successful, and WT TLR7 expression restored responses to TLR7 agonists (after removal of the inhibitor) (Fig. 2F, Fig. S4C). Hemizygosity for LOF *TLR7* alleles thus abolished responses to TLR7 stimulation in EBV-B cells, a phenotype that was rescued by WT TLR7 expression. Collectively, these findings further suggest that XR TLR7 deficiency is a genetic etiology of severe/critical COVID-19 pneumonia.

### The TLR7-mutated patients’ myeloid cells, including pDCs, do not respond to TLR7 agonists

**Fig. 3:**
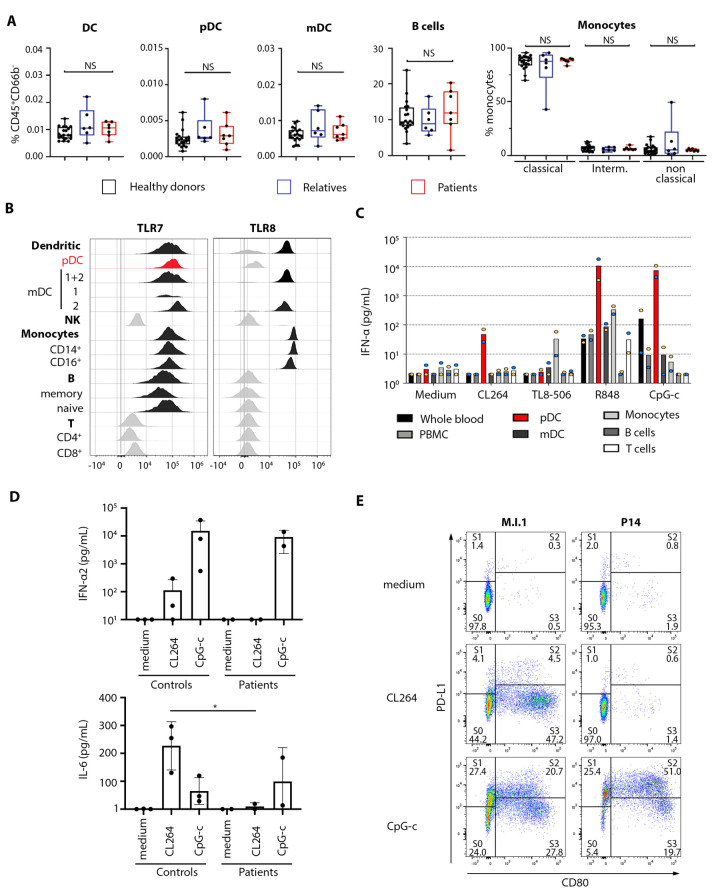
**Type I IFN responses to TLR7 agonist in TLR7-deficient pDCs and leukocytes. (A**) Frequencies of five leukocyte subsets in whole blood, determined by CyTOF. Healthy donors (black rectangles), relatives not carrying deleterious *TLR7* alleles (blue rectangles) and hemizygous *TLR7* variant carriers (red rectangles) are depicted. (**B**) TLR7 and TLR8 expression in different leukocyte subsets, determined by flow cytometry for the healthy control (C1). The result for another healthy control (C2) is shown in Figure S5C. Gating strategy for the classification in each cell subset is shown in Data file S6. (**C**) IFN-α production in purified leukocyte subsets from two healthy donors (blue or yellow dot) with and without stimulation with various TLR7, 8, or 9 agonists (1 μg/mL CL264, 100 ng/mL TL8-506, 1 μg/mL R848, or 2 μM CpG-c) for 24 hours. The y-axis shows IFN-α production on a logarithmic scale. The red bar corresponds to pDCs. (**D**) pDCs isolated from healthy donors and TLR7-deficient patients (P8, P14) were either left untreated (medium) or were stimulated with CL264 or CpG-c, and the production of IFN-α2 and IL-6 was assessed with CBAs on the supernatant. (**E**) Dotplot showing pDC diversification into subsets S1, S2, and S3 from magnetically sorted blood. pDCs from a TLR7-deficient patient (P14) and a healthy relative (M.I.1) were cultured for 24 hours with medium alone or with 1 μg/mL CL264 or 2 μM CpG-c. Statistical tests were performed using unpaired two-sample *t* test (*: P < 0.05).

Human TLR7 is known to be expressed and functional only in leukocyte subsets: plasmacytoid and classical dendritic cells (pDCs and mDCs), monocytes (classical, intermediate, and non-classical), and B cells (*27*, *32*, *40*). TLR8 is expressed in mDCs but not pDCs, monocytes but not B cells, and neutrophils (unlike TLR7) (*27*, *32*, *40*). Neither *TLR7* nor *TLR8* mRNAs have been detected in the lung or pulmonary epithelial cells (*41*). Deep immunophenotyping by CyTOF in seven patients with TLR7 deficiency revealed no major abnormalities in 18 peripheral blood leukocyte subsets, including pDCs, mDCs, monocytes, and B cells (Fig. 3A, Fig. S5A). We previously reported inherited IRF7 deficiency in a child with critical influenza pneumonia (*5*) and two unrelated adults with critical COVID-19 pneumonia (*8*). This defect disrupts the amplification of type I IFNs in all cell types, including pDCs, which are normally the main producers of type I IFN upon blood cell stimulation with TLR7 agonists or viruses, due to their constitutive expression of IRF7 (*27*, *42*–*44*). We hypothesized that TLR7 deficiency in pDCs impairs the production of type I IFN by these cells in response to ssRNA. We confirmed that TLR7 was expressed on pDCs, and that TLR8 was not (Fig. 3B, S5B, S5C). We measured the production of type I IFNs by purified leukocyte subsets (pDCs, mDCs, monocytes, B cells, T cells), in response to TLR7, TLR8 and TLR9 agonists (Fig. 3C, Fig. S5D). We confirmed that pDCs produced 100-1,000 times more type I IFN per cell than other leukocyte subsets upon TLR7 stimulation (Fig. 3C, Fig. S5D). We purified pDCs from P8 and P14 and analyzed their production of type I IFNs in response to CL264 and class C CpG oligonucleotide (CpG-c), relative to that of pDCs from healthy relatives, using a cytometric bead array (CBA) (Fig. 3D). pDCs from P8 and P14 did not produce type I IFNs (or IL-6) upon stimulation with a TLR7 agonist, whereas they responded to a TLR9 agonist (Fig. 3D). Moreover, agonist-induced up-regulation of PD-L1 and CD80 defines the maturation of pDCs into the S1 (PD-L1^high^/CD80^low^), S2 (PD-L1^high^/CD80^high^), and S3 (PD-L1^low^/CD80^high^) subsets (*45*). This maturation was not observed in the pDCs of P8 and P14, but was detected in the pDCs of healthy relatives and controls (Fig. 3E, Fig. S5E). Thus, pDCs from patients with *TLR7* mutations do not respond to TLR7 agonists in terms of maturation into specialized subsets and type I IFN production.

### The TLR7-deficient patients’ pDCs respond poorly to SARS-CoV-2

**Fig. 4:**
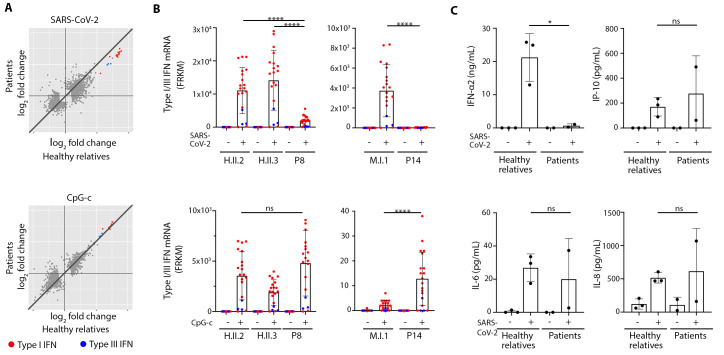
Type I IFN responses to SARS-CoV-2 infection in TLR7-deficient pDCs. (**A**) pDCs isolated from healthy relatives and TLR7-deficient patients (P8, P14) were either left untreated or were infected with SARS-CoV-2 for 24 hours. RNA profiles were then determined by RNA-seq. Genes with expression >2.0-fold higher or lower in controls after stimulation or infection are plotted as the fold-change in expression. (**B**) Induction of the type I and III IFN genes from (A) infected with SARS-CoV-2 for 24 hours (top) or stimulated with CpG-c (bottom). (**C**) pDCs isolated from healthy relatives and TLR7-deficient patients (P8, P14) were either left untreated or were infected with SARS-CoV-2 for 24 hours and the production of IFN-α2, IP-10, IL-6 and IL-8 was measured with CBAs on the supernatant. Statistical tests were performed using unpaired two-sample *t* test (*: P < 0.05, ****: P < 0.0001, ns: not significant).

A plausible mechanism accounting for the severity of COVID-19 in TLR7-deficient patients is the impairment of type I IFN production by pDCs upon stimulation with SARS-CoV-2, which can enter these cells, but cannot replicate productively within them (*45*, *46*). Indeed, we previously showed that the activation of human pDCs by SARS-CoV-2 depends on IRAK4 and UNC-93B, but not TLR3 (*45*). We tested the hypothesis that TLR7 is an essential pDC sensor of SARS-CoV-2, upstream from IRAK4 and UNC-93B, by infecting pDCs and pDC-depleted leukocytes from healthy controls and TLR7-deficient patients with SARS-CoV-2 for 24 hours. Control pDC-depleted leukocytes infected with SARS-CoV-2 displayed no significant up- or down-regulation of gene expression (Fig. S6A). By contrast, transcriptomic analysis showed a strong up-regulation of the type I IFN transcriptional module in pDCs from healthy controls, which was greatly reduced in pDCs from TLR7-deficient patients (Fig. 4A). Induction of the 17 type I *IFN* genes in pDCs from TLR7-deficient patients was 10 to 100 times weaker than that in pDCs from healthy individuals (Fig. 4B, S6B). We also analyzed the functional specialization of pDC subsets (S1-, S2-, and S3-pDC subsets) in response to SARS-CoV-2 activation (*45*, *47*). pDCs from P14 cultured with SARS-CoV-2 for 24 hours displayed abnormally low levels of maturation into the S1-subset —the pDC subset principally responsible for IFN-α production upon SARS-CoV-2 infection (Fig. S6C). Finally, we evaluated the amount of type I IFNs secreted by SARS-CoV-2-infected pDCs. All 13 individual IFN-α forms were produced in significantly smaller amounts by TLR7-deficient pDCs than by control pDCs (Fig. 4C, S6D). However, IFN-α production by TLR7-deficient pDCs upon SARS-CoV-2 infection was impaired, but not entirely abolished, as in UNC-93B- or IRF7-deficient pDCs (*8*, *45*), implying that there are also TLR7-independent sensors of SARS-CoV-2 in pDCs and suggesting that TLR9 is involved. The TLR7-deficient pDCs’ normal response to TLR9 agonists (Fig. 3D, 4A, 4B, S6D) is consistent with this hypothesis, while also suggesting that genetic or epigenetic variations of TLR9 responses may contribute to the apparently age-dependent penetrance of TLR7 deficiency. Thus, SARS-CoV-2 triggers type I IFN induction in pDCs in a manner that is dependent on TLR7, but not exclusively so. As pDCs are normally the main leukocytes producing type I IFN in such conditions, and type I IFN is essential for protective immunity to SARS-CoV-2 (*8*, *9*), these findings suggest that XR TLR7 deficiency underlies critical or severe COVID-19 pneumonia by disrupting TLR7-and pDC-dependent type I IFN production.

## DISCUSSION

We report XR TLR7 deficiency as a genetic etiology of severe/critical COVID-19 pneumonia in 20 unrelated male patients, aged 7 to 71 years, from seven countries. Only one of these 20 patients (5%) was older than 60 years, consistent with our previous observation that only five of 23 patients (21.7%) with inborn errors of TLR3-dependent type I IFN immunity were older than 60 years (*8*). This suggests that these genetic defects are mostly found in the youngest patients. This contrasts with the situation for auto-Abs against type I IFNs, which are found mostly in patients over the age of 60 years (*8*, *9*) (*79*). Importantly, patients with these auto-Abs do not overlap with those bearing inborn errors of TLR3- or TLR7-dependent type I IFNs. TLR7-deficient patients accounted for about 1.8% of the unrelated male patients with critical COVID-19 pneumonia below the age of 60 years in our cohort and accounted for 1.3% of the total cohort. This proportion remained around the same when severe COVID-19 pneumonia was also taken into account (1.7% males below 60 years; 1.2% of all the male patients in the total cohort). We also found that six of the 12 previously reported patients with a *TLR7* variant had TLR7 deficiency (*33*, *34*). It would be interesting to test experimentally the undisclosed *TLR7* variants reported to be enriched in another study (*48*). Our discovery provides an explanation for the higher risk of severe and critical disease in men than in women under the age of 60 years, complementing our previous observation of a much higher frequency of neutralizing auto-Abs against type I IFNs in men than in women with critical COVID-19 pneumonia for patients over the age of 60 years (*9*).

Previous reports of patients with critical COVID-19 pneumonia due to inborn errors of TLR3-dependent type I IFN immunity (*8*), including autosomal recessive IRF7 or IFNAR1 deficiency (*5*, *6*), or due to auto-Abs neutralizing type I IFNs (*9*, *11*–*14*, *16*, *17*), strongly suggest that critical disease in TLR7-deficient patients is a consequence of impaired type I IFN production upon SARS-CoV-2 infection. The absence of biochemically deleterious X-linked *TLR8* variants in our cohort of patients suggests that TLR8 is not essential for host defense against SARS-CoV-2. This is consistent with the modest capacity of TLR8 to induce type I IFN and its lack of expression on pDCs (*27*), and with the inflammatory phenotype of TLR8 gain-of-function mutations, which do not underlie a type I interferonopathy (*29*–*31*). Patients with inherited IRAK4 or MyD88 deficiency, whose cells do not respond to the stimulation of IL-1Rs and TLRs other than TLR3, including TLR7, have not been reported to display any severe viral illness over the almost 20 years since the discovery of IRAK-4 deficiency (*49*–*52*). Moreover, UNC-93B-deficient pDCs produced normal amounts of type I IFN in response to seasonal influenza virus (*5*). This was intriguing, as strong negative selection operates at the human *TLR7, TLR8,* and *TLR9* loci (*49*, *53*). Our study provides an answer to this riddle, by establishing that TLR7 is essential for protective immunity to SARS-CoV-2. Patients with IRAK4, MyD88, or UNC93B deficiency are now predicted to be vulnerable to SARS-CoV-2 (*54*–*56*). Critical COVID-19 and seasonal influenza can be caused by inborn errors of TLR3-dependent type I IFN immunity (*5*–*8*), but susceptibility to these infections is not allelic at the *TLR7* locus. It is, nevertheless, tempting to speculate that TLR7 might also be essential for host defense against more virulent, pandemic viruses, including both coronaviruses and influenza viruses.

Through the discovery of the essential nature of TLR7 for the induction of type I IFN in response to SARS-CoV-2, our study also reveals the essential function of human pDCs in host defense. The constitutively high levels of IRF7 in these cells make them the most potent producers of type I IFN in the blood, and perhaps in the entire human body, and this has long suggested a possible key role in antiviral immunity (*25*). However, the essential and redundant roles of this leukocyte subset have yet to be determined, in the absence of human pDC-specific deficiencies causally underlying a clinical phenotype. It has long been suspected, but never proved, that pDCs are essential for host defense in natural conditions (*26*, *57*–*59*). Inherited IRF7 deficiency, which underlies critical influenza or COVID-19 pneumonia, disrupts the production of type I IFNs not only by pDCs (*5*, *8*), but also by all other cell types, including pulmonary epithelial cells (*5*). Likewise, patients with GATA2 deficiency, who are prone to critical influenza (*60*), lack pDCs, but these patients also lack many other blood cell subsets (*61*–*64*). Inherited IFNAR1 deficiency underlies critical COVID-19 probably due to its broad cellular impact (*5*, *6*, *8*). By contrast, inborn errors of the TLR3 pathway underlie critical influenza or COVID-19 pneumonia by impairing the production of type I IFNs by cells other than pDCs, such as pulmonary epithelial cells (*5*–*8*, *65*). Our study indicates that pulmonary epithelial cells are not sufficient for host defense against SARS-CoV-2, as these cells do not express TLR7. Inborn errors of TLR7 are pathogenic by impairing the production of type I IFNs by blood pDCs, which are unique in their production of large amounts of both TLR7 and IRF7 (*66*, *67*). pDCs express other viral sensors, including TLR9 (for DNA), MDA5 and RIG-I (for dsRNA) (*68*), but TLR7 deficiency impairs their capacity to produce large enough amounts of type I IFN in response to SARS-CoV-2 in the respiratory tract. Overall, by disrupting pDC-dependent type I IFN production, XR TLR7 deficiency accounts for at least 1% of cases of life-threatening COVID-19 pneumonia in men under 60 years.

## MATERIALS AND METHODS

### Study design

We searched for X-linked inborn errors of immunity in male patients with critical SARS-CoV-2 pneumonia. We screened our WES database of 1,202 male patients with critical SARS-CoV-2 pneumonia (‘patients’) and 331 male subjects with asymptomatic or paucisymptomatic infection (‘controls’). We tested the association of X-linked genes with critical SARS-CoV-2 pneumonia using a Firth bias-corrected logistic regression model including the first five principal components of the PCA to account for the ethnic heterogeneity of the cohorts and age in years. We then tested the activity of *TLR7* variants in transduced cell lines and of *TLR7* genotypes in hemizygous patients’ cell lines. Lastly, we tested the patients’ pDCs for their response to both TLR7 agonists and SARS-CoV-2.

### Cohort recruitment and consent

This study included 1,202 male patients with life-threatening COVID-19 pneumonia, defined as patients with pneumonia who developed critical disease, whether pulmonary with high-flow oxygen (> 6L/min) or mechanical ventilation (CPAP, BIPAP, intubation), septic shock, or any other type of organ damage requiring ICU admission. This study also included patients with severe COVID-19 pneumonia, defined as hospitalized patients with pneumonia that required low-flow oxygen (<6L/min); moderate COVID-19 pneumonia, defined as patients with pneumonia but did not require oxygen therapy; and mild COVID-19, defined as patients with mild upper respiratory symptoms but without pneumonia. Patients who developed Kawasaki-like syndrome were excluded. The age of the patients ranged from 0.5-99 years, with a mean age of 52.9 years (SD 16.4 years). Asymptomatic or paucisymptomatic individuals (*n*= 331) were recruited on the basis of positive PCR or serological tests for SARS-CoV-2 in the absence of symptoms. These individuals were close contacts of patients or were recruited after clinical screening. The age of the asymptomatic or paucisymptomatic individuals ranged from 1.3-102 years, with a mean age of 38.7 years (SD: 17.2 years).

All the enrolled subjects provided written informed consent and were collected through protocols conforming to local ethics requirements. For patients enrolled in the French COVID cohort (clinicaltrials.gov NCT04262921), ethics approval was obtained from the CPP IDF VI (ID RCB: 2020-A00256-33) or the Ethics Committee of Erasme Hospital (P2020/203). For subjects enrolled in the COV-Contact study (clinicaltrials.gov NCT04259892), ethics approval was obtained from the CPP IDF VI (ID RCB: 2020-A00280-39). For patients enrolled in the Italian cohort, ethics approval was obtained from the University of Milano-Bicocca School of Medicine, San Gerardo Hospital, Monza – Ethics Committee of the National Institute of Infectious Diseases Lazzaro Spallanzani (84/2020) (Italy), and the Comitato Etico Provinciale (NP 4000 – Studio CORONAlab). STORM-Health care workers were enrolled in the STudio OsseRvazionale sullo screening dei lavoratori ospedalieri per COVID-19 (STORM-HCW) study, with approval from the local IRB obtained on June 18, 2020. Patients and relatives from San Raffaele Hospital (Milan) were enrolled in protocols COVID-BioB/Gene-COVID and, for additional studies, TIGET-06, which were approved by local ethical committee. For patients enrolled in Spain, the study was approved by the Committee for Ethical Research of the Infanta Leonor University Hospital, code 008-20, Committee for Ethical Research of the University Hospital 12 de Octubre, code 16/368 and the Bellvitge University Hospital code PR127/20, the University Hospital of Gran Canaria Dr. Negrín code 2020-200-1 COVID-19 and the Vall d’Hebron University Hospital, code PR(AMI)388/2016. Anonymized samples were sequenced at the NIAID through USUHS/TAGC under non-human subject research conditions; no additional IRB consent was required at the NIH. For patients enrolled in the Swedish COVID cohort, ethics approval was obtained from the Swedish Ethical Review Agency (2020-01911 05).

### Next-generation sequencing

Genomic DNA was extracted from whole blood. For the 1,533 patients included, the whole exome (*n*=1035) or whole genome (*n*=498) was sequenced at several sequencing centers, including the Genomics Core Facility of the Imagine Institute (Paris, France), the Yale Center for Genome Analysis (USA), the New-York Genome Center (NY, USA), and the American Genome Center (TAGC, USUHS, Bethesda, USA), and the Genomics Division-ITER of the Canarian Health System sequencing hub (Canary Islands, Spain).

For WES, libraries were generated with the Twist Bioscience kit (Twist Human Core Exome Kit), the xGen Exome Research Panel from Integrated DNA Technologies (IDT xGen), the Agilent SureSelect V7 kit or the SeqCap EZ MedExome kit from Roche, and the Nextera Flex for Enrichment-Exome kit (Illumina). Massively parallel sequencing was performed on a HiSeq4000 or NovaSeq6000 system (Illumina). For WES analysis performed at CNAG Barcelona, Spain, capture was performed with the SeqCap EZ Human Exome Kit v3.0 (Roche Nimblegen, USA) and 100-bp paired-end read sequences were obtained on a HiSeq 2000-4000 platform (Illumina, Inc. USA). For the OSR Italian cohort, WES was performed with the Agilent SureSelect V7 kit on a NovaSeq6000 system (Illumina).

For WGS on patients of the Italian cohort (TAGC), genomic DNA samples were dispensed into the wells of a Covaris 96 microTUBE plate (1,000 ng per well) and sheared with the Covaris LE220 Focused-ultrasonicator, at settings targeting a peak size of 410 bp (t:78; Duty:18; PIP:450; 200 cycles). Sequencing libraries were generated from fragmented DNA with the Illumina TruSeq DNA PCR-Free HT Library Preparation Kit, with minor modifications for automation (Hamilton STAR Liquid Handling System), with IDT for Illumina TruSeq DNA UD Index (96 indices, 96 samples) adapters. Library size distribution was assessed and the absence of free adapters or adapter dimers was checked by automated capillary gel electrophoresis (Advanced Analytical Fragment Analyzer). Library concentration was determined by qPCR with the KAPA qPCR Quantification Kit (Roche Light Cycler 480 Instrument II). Sequencing libraries were normalized and combined as 24-plex pools and quantified as above, before dilution to 2.9 nM and sequencing on an Illumina NovaSeq 6000 with the S4 Reagent Kit (300 cycles) and 151+8+8+151 cycle run parameters. Primary sequencing data were demultiplexed with the Illumina HAS2.2 pipeline and sample-level quality control was performed for base quality, coverage, duplicates and contamination (FREEMIX < 0.05 by VerifyBamID). For patients enrolled in the Swedish COVID cohort, sequencing was performed at the Clinical Genomics Stockholm unit of the SciLifeLab (Stockholm, Sweden).

We used the Genome Analysis Software Kit (GATK) (version 3.4-46 or 4) best-practice pipeline to analyze our WES data (*69*). We aligned the reads obtained with the human reference genome (hg19), using the maximum exact matches algorithm in the Burrows–Wheeler Aligner (BWA) (*70*). PCR duplicates were removed with Picard tools (picard.sourceforge.net). The GATK base quality score recalibrator was applied to correct sequencing artifacts. Genotyping was performed with GATK GenotypeGVCFs in the interval intersecting all the capture kits ± 50 bp. Sample genotypes with a coverage < 8X, a genotype quality (GQ) < 20, or a ratio of reads for the less covered allele (reference or variant allele) over the total number of reads covering the position (minor read ratio, MRR) < 20% were filtered out. We filtered out variant sites (i) with a call rate <50% in gnomAD genomes and exomes, (ii) a non-PASS filter in the gnomAD database, (iii) falling in low-complexity or decoy regions, (iv) that were multi-allelic with more than four alleles, (v) with more than 20% missing genotypes in our cohort, and (vi) spanning more than 20 nucleotides. Variant effects were predicted with the Ensembl Variant Effect Predictor (VEP) (*71*) and the Ensembl GRCh37.75 reference database, retaining the most deleterious annotation obtained from Ensembl canonical transcripts overlapping with RefSeq transcripts.

### Statistical analysis

We performed an enrichment analysis focusing on X chromosome genes on our cohort of 1,202 male patients with life-threatening COVID-19 pneumonia without known inborn errors of TLR3- and IRF7-dependent type I IFN immunity (*8*) and without neutralizing auto-Abs against type I IFNs (*9*), and 331 male individuals with asymptomatic or paucisymptomatic infection (Table S1). We considered variants that were predicted to be loss-of-function or missense, with a MAF below 0.0001 (gnomAD v2.1.1). We compared the proportion of patients and controls carrying at least one non-synonymous using the Firth bias-corrected logistic likelihood ratio test implemented in EPACTS (https://genome.sph.umich.edu/wiki/EPACTS) extended to gene based enrichment analysis. In Firth’s regression, a penalty term is placed on the standard maximum likelihood function used to estimate parameters of a logistic regression model (*18*). Firth’s can handle genes with no carriers among cases or controls. With no covariates, this corresponds to adding 0.5 in every cell of a 2x2 table of allele counts versus case-control status. We accounted for the ethnic heterogeneity of the cohorts by including the first five principal components of the PCA in the Firth’s logistic regression model. Analyses were also adjusted for age in years. We checked that our adjusted burden test was well-calibrated by also performing an analysis of enrichment in rare (MAF < 0.0001) synonymous variants. PCA was performed with Plink v1.9 software on whole-exome and whole-genome sequencing data, with the 1000 Genomes (1kG) Project phase 3 public database as a reference, using 18,917 exonic variants with a minor allele frequency > 0.01 and a call rate > 0.99.

### Cell culture

EBV-B cell lines derived from the patients were grown in complete RPMI 1640 (Life Technologies) supplemented with 10% heat-inactivated fetal bovine serum (FBS). HEK293T cells, derived from the human embryonic kidney 293 cell line, which expresses a mutant version of the SV40 large T antigen, were grown in complete DMEM (Life Technologies) supplemented with 10% FBS. Cells were incubated at 37°C in the presence of 5% CO_2_.

### Expression vectors and transfection experiments

All the *TLR7* variants in our analysis were generated by site-directed mutagenesis (Data file S4). The WT or variant alleles were re-introduced into a Myc-DDK-pCMV6 vector (Origene). HEK293T cells, which have no endogenous TLR7 or TLR8 expression, were transfected with the Myc-DDK-pCMV6 vector, empty or containing the WT or a variant allele, in the presence of X-tremeGENE 9 DNA Transfection Reagent (Sigma-Aldrich), according to the manufacturer’s instructions.

### Western blotting

For whole-cell extracts, the cells were lysed by incubation in the following buffer (50 mM Tris-HCl, pH 8.0, 150 mM NaCl, 1% NP40), supplemented with a mixture of protease inhibitors (Sigma-Aldrich), for 30 min at 4°C. The lysates were then centrifuged at 21,000 x *g* for 20 min at 4°C. The supernatants were processed directly for Western blotting. Western blotting was performed on 10 μg of total extract from transfected HEK293T cells, with monoclonal antibodies specific for the leucine-rich repeats to the N terminus within the human TLR7 protein (Cell Signaling Technology; clone, D7), or for amino-acid 1,000 to the C terminus with the human TLR7 protein (Abcam; clone, EPR2088(2)).

### Luciferase reporter assay

HEK293T cells, which have no endogenous *TLR7* expression, were transfected with the pCMV6 vector bearing wild-type or variant *TLR7* (50 ng), the reporter construct pGL4.32 (100 ng), and an expression vector for *Renilla* luciferase (10 ng), with the X-tremeGENE 9 DNA Transfection Reagent kit (Sigma-Aldrich). The pGL4.32 [luc2P/NF-κB-RE/Hygo] (Promega) reporter vector contains five copies of the NF-κB-responsive element (NF-κB-RE) linked to the luciferase reporter gene *luc2P*. After 24 hours, the transfected cells were left unstimulated or were stimulated with 1 μg/mL R848 (Resquimod), for activation via TLR7/8 (Invivogen), or 5 μg/mL R837 (Imiquimod) (Invivogen), or 5 μg/mL CL264 (Invivogen), human TLR7-specific agonists, for 24 hours. Relative luciferase activity was then determined by normalizing the values against the firefly:*Renilla* luciferase signal ratio.

### RNA extraction and reverse transcription-quantitative PCR (RT-qPCR)

Total RNA was extracted with the RNeasy Mini Kit (Qiagen), according to the manufacturer’s instructions. Reverse transcription was performed on 1 μg of RNA with random primers and the SuperScript^®^ III reverse transcriptase (Invitrogen), according to the manufacturer’s protocol. Quantitative PCR was then performed with the TaqMan Fast Universal PCR Master Mix (2X) and the FAM-MGB *Taq*Man *TNF* exons 1-2 (Hs99999-43_m1) probes. The VIC-TAMRA probe for *GUSB* (Applied Biosystems, Cat: 4310888E) was used as an endogenous control. Real-time PCR amplification was monitored with the 7500 Fast Real-Time PCR System (Applied Biosystems). Relative expression levels were determined according to the ΔCt method.

### ELISA analysis of TNF production in EBV-B cells

ELISA was performed as previously described (*50*). We suspended 1x10^6^ EBV-B cells per well in RPMI 1640 supplemented with 10% FBS. The cells were activated by incubation with 1 μg/mL R848, and 5 μg/mL imiquimod for 24 hours. The supernatants were harvested after 24 hours of activation. ELISA determinations of TNF in cell culture supernatants were performed with a kit (Thermo Fisher Scientific), according to the manufacturer’s instructions.

### Stable transduction

The WT coding sequence of *TLR7* was inserted into pTRIP-CMV-puro-2A. For lentivirus production, HEK293T cells were transfected with 1.6 μg pTRIP-CMV-puro-2A-TLR7-WT (or Mutant: K684*), 0.2 μg pCMV-VSV-G (Addgene), 0.2 μg pHXB2 (NIH-AIDS Reagent 22 Program) and 1 μg psPAX2 (Addgene), with X-treme gene 9 (Roche), according to the manufacturer's instructions. Supernatants were harvested after 24 hours and 8 μg/mL protamine sulfate was added. The lentiviral suspension obtained was used to transduce 2x10^5^ EBV-B cells by spinoculation at 1,200 x *g* for 2 hours. The transduced cells were selected by incubation on medium containing 1 μg/mL puromycin for two days. The cells were then selected by incubation for a further two days on medium containing 2 μg/mL puromycin. During viral transduction, the cells were cultured with 5 μM IRAK4 inhibitor (PF06650833) (Bio-techne) to prevent cell death due to the overproduction of TLR7. Selected transduced cells were then stimulated with 1 μg/mL R848 or 5 μg/mL imiquimod for 24 hours without IRAK4 inhibitor. The supernatants were harvested after 24 hours of activation. ELISA determinations of TNF in cell culture supernatants were performed with a kit (Thermo Fisher Scientific), according to the manufacturer’s instructions.

### VirScan analysis

Patient serum was analyzed by VirScan in two independent experiments as previously described (*78*). Briefly, an oligonucleotide library encoding 56 amino acid peptides tiling across the genomes of 206 viral species was synthesized on a releasable DNA microarray and cloned into T7 phage. Patient serum containing 2 μg of IgG was added to the phage library, and immunoprecipitation was performed with Protein A and G beads. Enriched peptides were identified by PCR and Illumina sequencing of the peptide cassette from the immunoprecipitated phage.

### Deep immunophenotyping by mass cytometry (CyTOF)

CyTOF was performed on whole blood with the Maxpar Direct Immune Profiling Assay (Fluidigm), according to the manufacturer’s instructions. Cells were frozen at -80°C after overnight staining to eliminate dead cells, and acquisition was performed on a Helios machine (Fluidigm). All the samples were processed within 24 hours of sampling. Data analysis was performed with OMIQ software. Antibody information is listed in supplemental material (Data file S5).

### PBMC enrichment using MACS system

Blood were collected from two healthy individuals and separated by the concentration gradient method with Ficoll^®️^ Paque Plus (Cytiva). After isolations of PBMCs, leucocyte subset (T cell, B cell, monocyte, pDC, and mDC) were purified by negative selection using MACS beads system (Milteni Biotec). Cells were plated into a U-bottomed 96-well plate at a density of 2×10^4^ cells/well for T cells, B cells, monocytes, pDCs, or mDCs in 200 μL/well RPMI-1640 with GlutaMAX supplemented with 10% FBS or 10×10^4^ cells/well for whole blood and PBMCs. Cells were left unstimulated or stimulated with 1μg/mL CL264, 100ng/ml TL8-506 (Invivogen), 1μg/mL R848, 2μM CpG-c (Invivogen), or 12.5ng/ml PMA and 0.125μM ionomycin for 24 hours. The supernatants were harvested after 24 hours of activation. Cytokines production were determined by ELISA (IFN-α - PBL Assay Science, IFN-β- PBL Assay Science, IFN- λ1 (IL-29) - Invivogen, IFN-ω- Invitrogen or IL-8 - R&D SYSTEMS); according to the manufacturer’s instructions.

### Analysis for TLR7 and TLR8 expression pattern in peripheral blood mononuclear cells (PBMCs) by flow cytometry

Freshly thawed PBMCs from healthy donors were dispensed into a V-bottomed 96-well plate at a density of 1×10^6^ cells/well, in 200 μL PBS/well. In brief, cells were stained by incubation with the LIVE/DEAD fixable blue dead-cell staining kit (Thermo Fisher Scientific, 1:800) and FcR blocking reagent (Miltenyi Biotec, 1:25) on ice for 15 min. For surface staining, cells were incubated with anti-γδTCR-BUV611 (BD Biosciences, 1:50), anti-CD183-BV750 (BD Biosciences, 1:20), and anti-CD194-BUV615 (BD Biosciences, 1:20) antibodies on ice for 30 min in 0.1% BSA and 0.01% sodium azide in PBS. They were then incubated with anti-CD141-BB515 (BD Biosciences, 1:40), anti-CD57-FITC (Biolegend, 1:83), anti-TCR Vδ2-PerCP (Biolegend, 1:166), anti-TCR Vα7.2-PerCP/Cyanine5.5 (Biolegend, 1:40), anti-TCR Vδ1-PerCP-Vio 700 (Miltenyi Biotec, 1:100), anti-CD14-Spark Blue 550 (Biolegend, 1:40), anti-CD1c-Alexa Fluor 647 (Biolegend, 1:50), anti-CD38-APC/Fire 810 (Biolegend, 1:30), anti-CD27-APC-H7 (BD Biosciences, 1:50), anti-CD127-APC-R700 (BD Biosciences, 1:50), anti-CD19-Spark NIR 685 (Biolegend, 1:83), anti-CD45RA-BUV395 (BD Biosciences, 1:83), anti-CD16-BUV496 (BD Biosciences, 1:166), anti-CD11b-BUV563 (BD Biosciences, 1:100), anti-CD56-BUV737 (BD Biosciences, 1:83), anti-CD8-BUV805 (BD Biosciences, 1:83), anti-hMR1-BV421 (NIH tetramer facility, 1:100), anti-CD11c-BV480 (BD Biosciences, 1:40), anti-CD45-BV510 (Biolegend, 1:83), anti-CD33-BV570 (Biolegend, 1:83), anti-iNKT-BV605 (Biolegend, 1:25), anti-CD161-BV650 (BD Biosciences, 1:25), anti-CCR6-BV711 (Biolegend, 1:83), anti-CCR7- BV785 (Biolegend, 1:40), anti-CD3-Pacific Blue (Biolegend, 1:83), anti-CD20-Pacific Orange (Life Technologies, 1:50), anti-CD123-Super Bright 436 (Invitrogen, 1:40), anti-CD24-PE-Alexa Fluor 610 (Life Technologies, 1:25), anti-CD25-PE-Alexa Fluor 700 (Life Technologies, 1:25), anti-CD294-Biotin (Invitrogen, 1:50), anti-CD209-PE/Cyanine7 (Biolegend, 1:25), anti-CD117-PE/Dazzle 594 (Biolegend, 1:83), anti-HLA-DR-PE/Fire 810 (Biolegend, 1:50), and anti-CD4-cFluor^TM^ YG584 (Cytek, 1:83) antibodies on ice for at least 30 min. The cells were then washed and stained by incubation with streptavidin-PE/Cy5 (Biolegend, 1:3000) on ice for 30 min. The cells were then fixed and permeabilized for intracellular staining with anti-TLR7-PE (Invitrogen) and anti-TLR8-APC (Biolegend) antibodies, with the eBioscience Foxp3/Transcription Factor Staining Buffer Set (Invitrogen), according to the manufacturer’s instructions. The cells were then washed and acquired with a five-laser Cytek Aurora (Cytek) flow cytometer. Antibody clone information is added in a supplemental material (Data file S6).

### pDC activation

Freshly purified pDCs were cultured in 96-well plates at a concentration of 5 × 10^5^ cells per mL in the presence of medium alone (RPMI 1640 Medium with GlutaMAX, 10% FBS, 1% MEM NEAA, 1% sodium pyruvate, and 1% penicillin/streptomycin), CL264 (Invivogen, 1 μg/mL), or the SARS-CoV-2 primary strain 220_95 (*45*) at a multiplicity of infection (MOI) of 1. After 24 hours of culture, the pDC supernatant was collected for cytokine quantification, and the PDCs were collected for diversification assessment by flow cytometry. In some experiments, RNA was purified from the pDCs were analyzed by RNA-seq (see below).

### Flow cytometry analysis for human pDCs

For assessments of pDC diversification, cells were stained with Zombie Violet fixable viability dye (Biolegend), BV711 anti-CD123 (Biolegend, clone 6H6), PE anti-CD80 (BD, clone L307.4), and PerCP-efluor 710 anti-PD-L1 (eBioscience, clone MIH1) antibodies. Data were acquired with an LSR Fortessa (BD Biosciences) flow cytometer and analyzed with FlowJo software (Tree Star). Flow cytometry analyses were performed at the flow cytometry core facility of IRSL (Paris, France).

### RNA-Sequencing

We collected cells from five individuals in two families: one patient (P8) and two healthy controls (H.II.2, H.II.3) from family H, and one patient (P14) and one healthy control (M.I.1)) from family M. These cells were stimulated with three conditions: non-stimulation, SARS CoV-2, and CpG-c. Total RNA was extracted from pDC cells with RNeasy Micro kits (QIAGEN). RNA-Seq libraries were prepared with the Illumina SMART-Seq^®^ v4 PLUS Kit (TaKaRa) and sequenced on the Illumina NextSeq 4000 platform with single-end 75 bp configuration. The RNA-Seq fastq raw data were inspected with multiQC v1.10 (*72*) to ensure the high quality of data. The sequencing reads were mapped onto the human reference genome GRCh38 with STAR aligner v2.7 (*73*), and the mapped reads were then quantified to determine the gene-level read counts with featureCounts V2.0.2 (*74*) and GENCODE human gene annotation GRCh38.p13 (*75*). The gene-level read counts were normalized and log2-transformed by DESeq2 (*76*), to obtain the gene expression profile of all samples for differential expression analysis. The differential gene expression was analyzed by applying TMM normalization and gene-wise generalized linear model regression with edgeR (*77*). The genes displaying significant differential expression were selected on the basis of |log2-FoldChange| ≥ 2 and FDR ≤ 0.05. The gene-level read counts of IFN genes were transformed to RPKM (Reads Per Kilobase of transcript, per Million mapped reads) by our own scripts, to compare the IFN gene expression of different samples under different stimulations.

### Determination of secreted inflammatory cytokines

We measured the production, by pDCs, of IFN-α2, IL-8, IL-6, and IP-10, by determining the levels of these cytokines in culture supernatants with the BD cytometric bead array (CBA), according to the manufacturer’s protocol, with a limit of detection of 20 pg/mL. Acquisitions were performed on an LSR Fortessa (BD Biosciences) flow cytometer, and cytokine concentrations were determined with FCAP Array Software (BD Biosciences).

## Supplementary materials

immunology.sciencemag.org/cgi/content/full/6/62/eabl4348/DC1
- Figure S1; Ethnicity information and *TLR7* allele activity - Figure S2; Allele activity for the *TLR8* variants found in our cohort - Figure S3; VirScan analysis of specific anti-viral antibodies detected in patient sera - Figure S4; Levels of TNF induction in EBV-B cells derived from two patients with XR TLR7 deficiency - Figure S5; Analysis of peripheral blood mononuclear cells from TLR7-deficient men - Figure S6; Functional analysis in pDCs infected with SARS-CoV-2 - Table S1; Characteristics of the cohort of patients with life-threatening COVID-19 pneumonia and the control cohort of asymptomatic or paucisymptomatic individuals - Table S2; Statistical analysis of non-synonymous rare variants of *TLR7* and *TLR8* in our cohorts - Table S3; Summary of *TLR7* variants - Data file S1; Selection of genes on chromosome X with 5 or more hemizygous carriers (Excel file). - Data file S2; *TLR7* variant activity reported in this study, in previous studies and in gnomAD (Excel file). - Data file S3; TLR7-deficient patients with severe/critical COVID-19 in our cohort (clinical information, laboratory findings, and immunological findings) (Excel file). - Data file S4; Primer sequences for mutagenesis (Excel file). - Data file S5; Antibody information for CyTOF (Excel file). - Data file S6; Gating strategy and antibody clone information for 40 color immunophenotyping (Excel file). - Data file S7; Raw data files (Excel file).

## COVID Human Genetic Effort

Laurent Abel^1^, Alessandro Aiuti^2^, Saleh Al-Muhsen^3^, Fahd Al-Mulla^4^, Mark S. Anderson^5^, Evangelos Andreakos^6^, Andrés A. Arias^7^, Hagit Baris Feldman^8^, Alexandre Belot^9^, Catherine M. Biggs^10^, Dusan Bogunovic^11^, Alexandre Bolze^12^, Anastasiia Bondarenko^13^, Ahmed A. Bousfiha^14^, Petter Brodin^15^, Yenan Bryceson^16^, Carlos D. Bustamante^17^, Manish J. Butte^18^, Giorgio Casari^19^, Samya Chakravorty^20^, John Christodoulou^21^, Antonio Condino-Neto^22^, Stefan N. Constantinescu^23^, Megan A. Cooper^24^, Clifton L. Dalgard^25^, Murkesh Desai^26^, Beth A. Drolet^27^, Jamila El Baghdadi^28^, Sara Espinosa-Padilla^29^, Jacques Fellay^30^, Carlos Flores^31^, José Luis Franco^7^, Antoine Froidure^32^, Peter K. Gregersen^33^, Filomeen Haerynck^34^, David Hagin^35^, Rabih Halwani​^36^, Lennart Hammarström^37^, James R. Heath^38^, Sarah E. Henrickson^39^, Elena W.Y. Hsieh^40^, Eystein Husebye^41^, Kohsuke Imai^42^, Yuval Itan^43^, Erich D. Jarvis^44^, Timokratis Karamitros^45^, Kai Kisand^46^, Cheng-Lung Ku^47^, Yu-Lung Lau^48^, Yun Ling^49^, Carrie L. Lucas^50^, Tom Maniatis^51^, Davood Mansouri^52^, László Maródi^53^, Isabelle Meyts^54^, Joshua D. Milner^55^, Kristina Mironska^56^, Trine H. Mogensen^57^, Tomohiro Morio^58^, Lisa F.P. Ng^59^, Luigi D. Notarangelo^60^, Antonio Novelli^61^, Giuseppe Novelli^62^, Cliona O'Farrelly^63^, Satoshi Okada^64^, Tayfun Ozcelik^65^, Qiang Pan-Hammarström^37^, Rebeca Perez de Diego^66^, Anna M. Planas^67^, Carolina Prando^68^, Aurora Pujol^69^, Lluis Quintana-Murci^70^, Laurent Renia^59^, Igor Resnick^71^, Carlos Rodríguez-Gallego^72^, Vanessa Sancho-Shimizu^73^, Anna Sediva^74^, Mikko R.J. Seppänen^75^, Mohammed Shahrooei^76^, Anna Shcherbina^77^, Ondrej Slaby^78^, Andrew L. Snow^79^, Pere Soler-Palacín^80^, András N. Spaan^81^, Ivan Tancevski^82^, Stuart G. Tangye^83^, Ahmad Abou Tayoun^84^, Sathishkumar Ramaswamy^84^, Stuart E Turvey^85^, K M Furkan Uddin^86^, Mohammed J. Uddin^87^, Diederik van de Beek^88^, Donald C. Vinh^89^, Horst von Bernuth^90^, Mayana Zatz^91^, Pawel Zawadzki^92^, Helen C. Su^60^, Jean-Laurent Casanova^93^

^1^INSERM U1163, University of Paris, Imagine Institute, Paris, France. ^2^San Raffaele Telethon Institute for Gene Therapy, IRCCS Ospedale San Raffaele, and Vita Salute San Raffaele University, Milan, Italy. ^3^Immunology Research Laboratory, Department of Pediatrics, College of Medicine and King Saud University Medical City, King Saud University, Riyadh, Saudi Arabia. ^4^Dasman Diabetes Institute, Department of Genetics and Bioinformatics, Dasman, Kuwait. ^5^Diabetes Center, University of California San Francisco, San Francisco, CA, USA. ^6^Laboratory of Immunobiology, Center for Clinical, Experimental Surgery and Translational Research, Biomedical Research Foundation of the Academy of Athens, Athens, Greece. ^7^Universidad de Antioquia, Group of Primary Immunodeficiencies, Antioquia UdeA, Medellín, Colombia. ^8^The Genetics Institute, Tel Aviv Sourasky Medical Center and Sackler Faculty of Medicine, Tel Aviv University, Tel Aviv, Israel. ^9^Pediatric Nephrology, Rheumatology, Dermatology, HFME, Hospices Civils de Lyon, National Referee Centre RAISE, and INSERM U1111, Université de Lyon, Lyon, France. ^10^Department of Pediatrics, British Columbia Children’s Hospital, The University of British Columbia, Vancouver, BC, Canada ^11^Icahn School of Medicine at Mount Sinai, New York, NY, USA. ^12^Helix, San Mateo, CA, USA. ^13^Shupyk National Medical Academy for Postgraduate Education, Kiev, Ukraine. ^14^Clinical Immunology Unit, Department of Pediatric Infectious Disease, CHU Ibn Rushd and LICIA, Laboratoire d'Immunologie Clinique, Inflammation et Allergie, Faculty of Medicine and Pharmacy, Hassan II University, Casablanca, Morocco. ^15^SciLifeLab, Department Of Women’s and Children’s Health, Karolinska Institutet, Stockholm, Sweden ^16^Department of Medicine, Center for Hematology and Regenerative Medicine, Karolinska Institutet, Stockholm, Sweden. ^17^Stanford University, Stanford, CA, USA. ^18^Division of Immunology, Allergy, and Rheumatology, Department of Pediatrics and the Department of Microbiology, Immunology, and Molecular Genetics, University of California, Los Angeles, CA, USA. ^19^Clinical Genomics, IRCCS San Raffaele Scientific Institute and Vita-Salute San Raffaele University, Milan, Italy ^20^Department of Pediatrics and Children’s Healthcare of Atlanta, Emory University, Atlanta, GA, USA. ^21^Murdoch Children's Research Institute and Department of Pediatrics, University of Melbourne, Australia ^22^Department of Immunology, Institute of Biomedical Sciences, University of São Paulo, São Paulo, Brazil. ^23^de Duve Institute and Ludwig Cancer Research, Brussels, Belgium ^24^Washington University School of Medicine, St. Louis, MO, USA. ^25^Department of Anatomy, Physiology & Genetics, Uniformed Services University of the Health Sciences, Bethesda, MD, USA. ^26^Bai Jerbai Wadia Hospital for Children, Mumbai, India. ^27^School of Medicine and Public Health, University of Wisconsin, Madison, WI, USA. ^28^Genetics Unit, Military Hospital Mohamed V, Rabat, Morocco. ^29^Instituto Nacional de Pediatria (National Institute of Pediatrics), Mexico City, Mexico. ^30^School of Life Sciences, Ecole Polytechnique Fédérale de Lausanne, Lausanne, Switzerland; Precision Medicine Unit, Lausanne University Hospital and University of Lausanne, Lausanne, Switzerland. ^31^Genomics Division, Instituto Tecnológico y de Energías Renovables (ITER), Santa Cruz de Tenerife, Spain; Research Unit, Hospital Universitario N.S. de Candelaria, Santa Cruz de Tenerife, Spain; Instituto de Tecnologías Biomédicas (ITB), Universidad de La Laguna, San Cristóbal de La Laguna, Spain; CIBER de Enfermedades Respiratorias, Instituto de Salud Carlos III, Madrid, Spain. ^32^Pulmonology Department, Cliniques Universitaires Saint-Luc ; Institut de Recherche Expérimentale et Clinique (IREC), Université Catholique de Louvain, Brussels, Belgium. ^33^Feinstein Institute for Medical Research, Northwell Health USA, Manhasset, NY, USA. ^34^Department of Pediatric Immunology and Pulmonology, Centre for Primary Immunodeficiency Ghent (CPIG), PID Research Laboratory, Jeffrey Model Diagnosis and Research Centre, Ghent University Hospital, Ghent, Belgium. ^35^The Genetics Institute Tel Aviv Sourasky Medical Center, Tel Aviv, Israel. ^36^Sharjah Institute of Medical Research, College of Medicine, University of Sharjah, Sharjah, United Arab Emirates. ^37^Department of Biosciences and Nutrition, Karolinska Institutet, Stockholm, Sweden. ^38^Institute for Systems Biology, Seattle, WA, USA. ^39^Department of Pediatrics, Division of Allergy Immunology, Children’s Hospital of Philadelphia, Philadelphia, PA, USA; Department of Microbiology, Perelman School of Medicine, University of Pennsylvania, Philadelphia, PA, USA ^40^Departments of Pediatrics, Immunology and Microbiology, University of Colorado, School of Medicine, Aurora, Colorado, USA ^41^Department of Medicine, Haukeland University Hospital, Bergen, Norway. ^42^Department of Community Pediatrics, Perinatal and Maternal Medicine, Tokyo Medical and Dental University (TMDU) ^43^Institute for Personalized Medicine, Icahn School of Medicine at Mount Sinai, New York, NY, USA; Department of Genetics and Genomic Sciences, Icahn School of Medicine at Mount Sinai, New York, NY, USA. ^44^Laboratory of Neurogenetics of Language and Howard Hughes Medical Institute, The Rockefeller University, New York, NY, USA. ^45^Bioinformatics and Applied Genomics Unit, Hellenic Pasteur Institute, Athens, Greece ^46^Molecular Pathology, Department of Biomedicine, Institute of Biomedicine and Translational Medicine, University of Tartu, Tartu Estonia. ^47^Chang Gung University, Taoyuan County, Taiwan. ^48^Department of Pediatrics & Adolescent Medicine, The University of Hong Kong, Hong Kong, China. ^49^Shanghai Public Health Clinical Center, Fudan University, Shanghai, China. ^50^Department of Immunobiology, Yale University School of Medicine, New Haven, CT, USA. ^51^Columbia University Zuckerman Institute, New York, NY ^52^Department of Clinical Immunology and Infectious Diseases, National Research Institute of Tuberculosis and Lung Diseases, The Clinical Tuberculosis and Epidemiology Research Center, National Research Institute of Tuberculosis and Lung Diseases (NRITLD), Masih Daneshvari Hospital, Shahid Beheshti University of Medical Sciences, Tehran, Iran. ^53^Primary Immunodeficiency Clinical Unit and Laboratory, Department of Dermatology, Venereology and Dermatooncology, Semmelweis University, Budapest, Hungary. ^54^Department of Pediatrics, University Hospitals Leuven; KU Leuven, Department of Microbiology, Immunology and Transplantation; Laboratory for Inborn Errors of Immunity, KU Leuven, Leuven, Belgium. ^55^Department of Pediatrics, Columbia University Irving Medical Center, New York, NY, USA. ^56^University Clinic for Children's Diseases, Department of Pediatric Immunology, Medical Faculty, University “St.Cyril and Methodij” Skopje, North Macedonia. ^57^Department of Biomedicine, Aarhus University, Aarhus, Denmark ^58^Tokyo Medical & Dental University Hospital, Tokyo, Japan. ^59^A*STAR Infectious Disease Labs, Agency for Science, Technology and Research, Singapore; Lee Kong Chian School of Medicine, Nanyang Technology University, Singapore. ^60^National Institute of Allergy and Infectious Diseases, National Institutes of Health, Bethesda, MD, USA. ^61^Laboratory of Medical Genetics, IRCCS Bambino Gesù Children’s Hospital, Rome, Italy. ^62^Department of Biomedicine and Prevention, Tor Vergata University of Rome, Rome, Italy. ^63^Comparative Immunology Group, School of Biochemistry and Immunology, Trinity Biomedical Sciences Institute, Trinity College Dublin, Ireland. ^64^Department of Pediatrics, Graduate School of Biomedical and Health Sciences, Hiroshima University, Hiroshima, Japan. ^65^Department of Molecular Biology and Genetics, Bilkent University, Bilkent - Ankara, Turkey. ^66^Laboratory of Immunogenetics of Human Diseases, Innate Immunity Group, IdiPAZ Institute for Health Research, La Paz Hospital, Madrid, Spain. ^67^IIBB-CSIC, IDIBAPS, Barcelona, Spain. ^68^Faculdades Pequeno Príncipe, Instituto de Pesquisa Pelé Pequeno Príncipe, Curitiba, Brazil. ^69^Neurometabolic Diseases Laboratory, Bellvitge Biomedical Research Institute (IDIBELL), L'Hospitalet de Llobregat, Barcelona, Spain; Catalan Institution of Research and Advanced Studies (ICREA), Barcelona, Spain; Center for Biomedical Research on Rare Diseases (CIBERER), ISCIII, Barcelona, Spain. ^70^Human Evolutionary Genetics Unit, CNRS U2000, Institut Pasteur, Paris, France; Human Genomics and Evolution, Collège de France, Paris, France. ^71^University Hospital St. Marina, Varna, Bulgaria. ^72^Department of Immunology, University Hospital of Gran Canaria Dr. Negrín, Canarian Health System, Las Palmas de Gran Canaria, Spain; Department of Clinical Sciences, University Fernando Pessoa Canarias, Las Palmas de Gran Canaria, Spain ^73^Department of Pediatric Infectious Diseases and Virology, Imperial College London, London, UK; Centre for Pediatrics and Child Health, Faculty of Medicine, Imperial College London, London, UK. ^74^Department of Immunology, Second Faculty of Medicine Charles University, V Uvalu, University Hospital in Motol, Prague, Czech Republic. ^75^Adult Immunodeficiency Unit, Infectious Diseases, Inflammation Center, University of Helsinki and Helsinki University Hospital, Helsinki, Finland; Rare Diseases Center and Pediatric Research Center, Children's Hospital, University of Helsinki and Helsinki University Hospital, Helsinki, Finland ^76^Saeed Pathobiology and Genetics Lab, Tehran, Iran; Department of Microbiology and Immunology, Clinical and Diagnostic Immunology, KU Leuven, Leuven, Belgium. ^77^Department of Immunology, Dmitry Rogachev National Medical Research Center of Pediatric Hematology, Oncology and Immunology, Moscow, Russia. ^78^Central European Institute of Technology & Department of Biology, Faculty of Medicine, Masaryk University, Brno, Czech Republic. ^79^Department of Pharmacology & Molecular Therapeutics, Uniformed Services University of the Health Sciences, Bethesda, MD, USA. ^80^Pediatric Infectious Diseases and Immunodeficiencies Unit, Vall d’Hebron Barcelona Hospital Campus, Barcelona, Spain. ^81^St. Giles Laboratory of Human Genetics of Infectious Diseases, Rockefeller Branch, The Rockefeller University, New York, NY, USA.; Department of Medical Microbiology, University Medical Center Utrecht, Utrecht, Netherlands ^82^Department of Internal Medicine II, Medical University of Innsbruck, Innsbruck, Austria. ^83^Garvan Institute of Medical Research, Darlinghurst, NSW, Australia; St Vincent’s Clinical School, Faculty of Medicine, UNSW Sydney, NSW, Australia. ^84^Al Jalila Children's Hospital, Dubai, UAE ^85^BC Children's Hospital, The University of British Columbia, Vancouver, Canada ^86^Centre for Precision Therapeutics, Genetic and Genomic Medicine Centre, NeuroGen Children Healthcare, Dhaka, Bangladesh; Holy Family Red Crescent Medical College, Dhaka, Bangladesh ^87^College of Medicine, Mohammed Bin Rashid University of Medicine and Health Sciences, Dubai, UAE; Cellular Intelligence (Ci) Lab, GenomeArc Inc., Toronto, ON, Canada ^88^Department of Neurology, Amsterdam Neuroscience, Amsterdam University Medical Center, University of Amsterdam, Amsterdam, The Netherlands. ^89^Department of Medicine, Division of Infectious Diseases, McGill University Health Centre, Montréal, Québec, Canada; Infectious Disease Susceptibility Program, Research Institute, McGill University Health Centre, Montréal, Québec, Canada. ^90^Department of Pediatric Pneumology, Immunology and Intensive Care, Charité Universitätsmedizin, Berlin University Hospital Center, Berlin, Germany; Labor Berlin GmbH, Department of Immunology, Berlin, Germany; Berlin Institutes of Health (BIH), Berlin-Brandenburg Center for Regenerative Therapies, Berlin, Germany. ^91^Biosciences Institute, University of São Paulo, São Paulo, Brazil. ^92^Molecular Biophysics Division, Faculty of Physics, A. Mickiewicz University, Poznań, Poland. ^93^The Rockefeller University & Howard Hughes Medical Institute, New York, NY, USA; Necker Hospital for Sick Children & INSERM, Paris, France.

## COVID-STORM Clinicians

Giuseppe Foti^1^, Giacomo Bellani^1^, Giuseppe Citerio^1^, Ernesto Contro^1^, Alberto Pesci^2^, Maria Grazia Valsecchi^3^, Marina Cazzaniga^4^

^1^Department of Emergency, Anesthesia and Intensive Care, School of Medicine and Surgery, University of Milano-Bicocca, San Gerardo Hospital, Monza, Italy. ^2^Department of Pneumology, School of Medicine and Surgery, University of Milano-Bicocca, San Gerardo Hospital, Monza, Italy. ^3^Center of Bioinformatics and Biostatistics, School of Medicine and Surgery, University of Milano-Bicocca, San Gerardo Hospital, Monza, Italy. ^4^Phase I Research Center, School of Medicine and Surgery, University of Milano-Bicocca, San Gerardo Hospital, Monza, Italy.

## COVID Clinicians

Jorge Abad^1^, Giulia Accordino^2^, Cristian Achille^3^, Sergio Aguilera-Albesa^4^, Aina Aguiló-Cucurull^5^, Alessandro AIUTI^6^, Esra Akyüz Özkan^7^, Ilad Alavi Darazam^8^, Jonathan Antonio Roblero Albisures^9^, Juan C Aldave^10^, Miquel Alfonso Ramos^11^, Taj Ali Khan^12^, Anna Aliberti^13^, Seyed Alireza Nadji^14^, Gulsum Alkan^15^, Suzan A. AlKhater^16^, Jerome Allardet-Servent^17^, Luis M Allende^18^, Rebeca ALONSO-ARIAS^19^, Mohammed S Alshahrani^20^, Laia Alsina^21^, Marie-Alexandra Alyanakian^22^, Blanca Amador Borrero^23^, Zahir Amoura^24^, Arnau Antolí^25^, Romain Arrestier^26^, Mélodie Aubart^27^, Teresa Auguet^28^, Iryna Avramenko^29^, Gökhan Aytekin^30^, Axelle Azot^31^, Seiamak Bahram^32^, Fanny Bajolle^33^, Fausto Baldanti^34^, Aurélie Baldolli^35^, Maite Ballester^36^, Hagit Baris Feldman^37^, Benoit Barrou^38^, Federica BARZAGH^6^, Sabrina Basso^39^, Gulsum Iclal BAYHAN^40^, Alexandre Belot^41^, Liliana BEZRODNIK^42^, Agurtzane Bilbao^43^, Geraldine Blanchard-Rohner^44^, Ignacio Blanco^45^, Adeline Blandinières^46^, Daniel Blázquez-Gamero^47^, Alexandre Bleibtreu^48^, Marketa Bloomfield^49^, Mireia Bolivar-Prados^50^, Anastasiia BONDARENKO^51^, Alessandro Borghesi^3^, Raphael Borie^52^, Elisabeth Botdhlo-Nevers^53^, Ahmed A Bousfiha^54^, Aurore Bousquet^55^, David Boutolleau^56^, Claire Bouvattier^57^, Oksana Boyarchuk^58^, Juliette Bravais^59^, M. Luisa Briones^60^, Marie-Eve Brunner^61^, Raffaele Bruno^62^, Maria Rita P Bueno^63^, Huda Bukhari^64^, Jacinta Bustamante^33^, Juan José Cáceres Agra^65^, Ruggero Capra^66^, Raphael Carapito^67^, Maria Carrabba^68^, Giorgio CASARI^6^, Carlos Casasnovas^69^, Marion Caseris^70^, Irene Cassaniti^34^, Martin Castelle^71^, Francesco Castelli^72^, Martín Castillo de Vera^73^, Mateus V Castro^63^, Emilie Catherinot^74^, Jale Bengi Celik^75^, Alessandro Ceschi^76^, Martin Chalumeau^77^, Bruno Charbit^78^, Matthew P. Cheng^79^, Père Clavé^50^, Bonaventura Clotet^80^, Anna Codina^81^, Yves Cohen^82^, Roger Colobran^83^, Cloé Comarmond^84^, Alain Combes^85^, Patrizia Comoli^39^, Angelo G Corsico^2^, Taner Coşkuner^86^, Aleksandar Cvetkovski^87^, Cyril Cyrus^88^, David Dalmau^89^, François Danion^90^, David Ross Darley^91^, Vincent Das^92^, Nicolas Dauby^93^, Stéphane Dauger^94^, Paul De Munter^95^, Loic de Pontual^96^, Amin Dehban^97^, Geoffroy Delplancq^98^, Alexandre Demoule^99^, Isabelle Desguerre^100^, Antonio Di Sabatino^101^, Jean-Luc Diehl^102^, Stephanie Dobbelaere^103^, Elena Domínguez-Garrido^104^, Clément Dubost^105^, Olov EKWALL^106^, Şefika Elmas Bozdemir^107^, Marwa H Elnagdy^108^, Melike Emiroglu^15^, Akifumi Endo^109^, Emine Hafize Erdeniz^110^, Selma Erol Aytekin^111^, Maria Pilar ETXART LASA^112^, Romain Euvrard^113^, Giovanna Fabio^68^, Laurence Faivre^114^, Antonin Falck^115^, Muriel Fartoukh^116^, Morgane Faure^117^, Miguel Fernandez Arquero^118^, Ricard Ferrer^119^, Jose Ferreres^120^, Carlos Flores^121^, Bruno Francois^122^, Victoria Fumadó^123^, Kitty S C Fung^124^, Francesca Fusco^125^, Alenka Gagro^126^, Blanca Garcia Solis^127^, Pascale Gaussem^128^, Zeynep GAYRETLI^129^, Juana Gil-Herrera^130^, Laurent Gilardin^131^, Audrey Giraud Gatineau^132^, Mònica Girona-Alarcón^133^, Karen Alejandra Cifuentes Godínez^134^, Jean-Christophe Goffard^135^, Nacho GONZALES^136^, Luis I Gonzalez-Granado^137^, Rafaela González-Montelongo^138^, Antoine Guerder^139^, Belgin Gülhan^140^, Victor Daniel Gumucio^141^, Leif Gunnar Hanitsch^142^, Jan Gunst^143^, Marta Gut^144^, Jérôme Hadjadj^145^, Filomeen Haerynck^146^, Rabih Halwani^147^, Lennart Hammarström^148^, Selda HANCERLI^149^, Tetyana Hariyan^150^, Nevin Hatipoglu^151^, Deniz Heppekcan^152^, Elisa Hernandez-Brito^153^, Po-ki Ho^154^, María Soledad Holanda-Peña^155^, Juan P Horcajada^156^, Sami Hraiech^157^, Linda Humbert^158^, Ivan F N Hung^159^, Alejandro D. Iglesias^160^, Antonio Íñigo-Campos^138^, Matthieu Jamme^161^, María Jesús Arranz^89^, Marie-Thérèse Jimeno^162^, Iolanda Jordan^133^, Saliha Kanık Yüksek^163^, Yalcin Burak Kara^164^, Aydın Karahan^165^, Adem KARBUZ^166^, Kadriye Kart Yasar^167^, Ozgur Kasapcopur^168^, Kenichi Kashimada^169^, Sevgi Keles^111^, Yasemin Kendir Demirkol^170^, Yasutoshi Kido^171^, Can KIZIL^172^, Ahmet Osman Kılıç^173^, Adam Klocperk^174^, Antonia Koutsoukou^175^, Zbigniew J. Król^176^, Hatem Ksouri^177^, Paul Kuentz^178^, Arthur M C Kwan^179^, Yat Wah M Kwan^180^, Janette S Y Kwok^181^, Jean-Christophe Lagier^182^, David S Y Lam^183^, Vicky Lampropoulou^184^, Fanny Lanternier^185^, Yu-Lung LAU^186^, Fleur Le Bourgeois^94^, Yee-Sin Leo^187^, Rafael Leon Lopez^188^, Daniel Leung^186^, Michael Levin^189^, Michael Levy^94^, Romain Lévy^33^, Zhi Li^78^, Daniele Lilleri^34^, Edson Jose Adrian Bolanos Lima^190^, Agnes Linglart^191^, Eduardo López-Collazo^192^, José M. Lorenzo-Salazar^138^, Céline Louapre^193^, Catherine Lubetzki^193^, Kwok-Cheung Lung^194^, Charles-Edouard Luyt^195^, David C Lye^196^, Cinthia MAGNONE^197^, Davood Mansouri^198^, Enrico Marchioni^199^, Carola Marioli^2^, Majid Marjani^200^, Laura MARQUES^201^, Jesus Marquez Pereira^202^, Andrea Martín-Nalda^203^, David Martínez Pueyo^204^, Javier Martinez-Picado^205^, Iciar Marzana^206^, Carmen Mata-Martínez^207^, Alexis Mathian^24^, Larissa RB Matos^63^, Gail V Matthews^208^, Julien Mayaux^209^, Raquel McLaughlin-Garcia^210^, Philippe Meersseman^211^, Jean-Louis Mège^212^, Armand Mekontso-Dessap^213^, Isabelle Melki^115^, Federica Meloni^2^, Jean-François Meritet^214^, Paolo Merlani^215^, Özge METIN AKCAN^216^, Isabelle Meyts^217^, Mehdi Mezidi^218^, Isabelle Migeotte^219^, Maude Millereux^220^, Matthieu Million^221^, Tristan Mirault^222^, Clotilde Mircher^223^, Mehdi Mirsaeidi^224^, Yoko Mizoguchi^225^, Bhavi P Modi^226^, Francesco Mojoli^13^, Elsa MONCOMBLE^227^, Abián Montesdeoca Melián^228^, Antonio Morales Martinez^229^, Francisco Morandeira^230^, Pierre-Emmanuel Morange^231^, Cléemence Mordacq^158^, Guillaume Morelle^232^, Stéphane J Mouly^233^, Adrián Muñoz-Barrera^138^, Cyril Nafati^234^, Shintaro Nagashima^235^, Yu Nakagama^171^, Bénédicte Neven^236^, João Farela Neves^237^, Lisa FP Ng^238^, Yuk-Yung Ng^239^, hubert Nielly^105^, Yeray Novoa Medina^210^, Esmeralda Nuñez Cuadros^240^, J. Gonzalo Ocejo-Vinyals^241^, Keisuke Okamoto^109^, Mehdi Oualha^33^, Amani Ouedrani^22^, Tayfun Özçelik^242^, Aslinur Ozkaya-Parlakay^140^, Michele Pagani^13^, Qiang Pan-Hammarström^148^, Maria Papadaki^243^, Christophe Parizot^209^, Philippe Parola^244^, Tiffany Pascreau^245^, Stéphane Paul^246^, Estela Paz-Artal^247^, Sigifredo Pedraza^248^, Nancy Carolina González Pellecer^134^, Silvia Pellegrini^249^, Rebeca Pérez de Diego^127^, Xosé Luis Pérez-Fernández^141^, Aurélien Philippe^250^, Quentin Philippot^116^, Adrien Picod^251^, Marc Pineton de Chambrun^85^, Antonio Piralla^34^, Laura Planas-Serra^252^, Dominique Ploin^253^, Julien Poissy^254^, Géraldine Poncelet^70^, Garyphallia Poulakou^175^, Marie S Pouletty^255^, Persia Pourshahnazari^256^, Jia Li Qiu-Chen^257^, Paul Quentric^209^, Thomas Rambaud^258^, Didier Raoult^212^, Violette RAOULT^259^, Anne-Sophie Rebillat^223^, Claire Redin^260^, Léa Resmini^261^, Pilar Ricart^262^, Jean-Christophe Richard^263^, Raúl Rigo-Bonnin^264^, Nadia rivet^46^, Jacques G Rivière^265^, Gemma Rocamora-Blanch^25^, Mathieu P RODERO^266^, Carlos Rodrigo^267^, Luis Antonio Rodriguez^190^, Carlos Rodriguez-Gallego^268^, Agustí Rodriguez-Palmero^269^, Carolina Soledad Romero^270^, Anya Rothenbuhler^271^, Damien Roux^272^, Nikoletta Rovina^175^, Flore Rozenberg^273^, Yvon Ruch^90^, Montse Ruiz^274^, Maria Yolanda Ruiz del Prado^275^, Juan Carlos Ruiz-Rodriguez^119^, Joan Sabater-Riera^141^, Kai Saks^276^, Maria Salagianni^184^, Oliver Sanchez^277^, Adrián Sánchez-Montalvá^278^, Silvia Sánchez-Ramón^279^, Laire Schidlowski^280^, Agatha Schluter^252^, Julien Schmidt^281^, Matthieu Schmidt^282^, Catharina Schuetz^283^, Cyril E Schweitzer^284^, Francesco Scolari^285^, Anna Sediva^286^, Luis Seijo^287^, Analia Gisela Seminario^42^, Damien Sene^23^, Piseth Seng^221^, Sevtap Senoglu^167^, Mikko Seppänen^288^, Alex Serra Llovich^289^, Mohammad Shahrooei^97^, Anna Shcherbina^290^, Virginie Siguret^291^, Eleni Siouti^292^, David M Smadja^293^, Nikaia Smith^78^, Ali Sobh^294^, Xavier Solanich^25^, Jordi Solé-Violán^295^, Catherine Soler^296^, Pere Soler-Palacín^297^, Betül Sözeri^86^, Giulia Maria Stella^2^, Yuriy Stepanovskiy^298^, Annabelle Stoclin^299^, Fabio Taccone^219^, Yacine Tandjaoui-Lambiotte^300^, Jean-Luc Taupin^301^, Simon J Tavernier^302^, Loreto Vidaur Tello^112^, Benjamin Terrier^303^, Guillaume Thiery^304^, Christian Thorball^260^, Karolina THORN^305^, Caroline Thumerelle^158^, Imran Tipu^306^, Martin Tolstrup^307^, Gabriele Tomasoni^308^, Julie Toubiana^77^, Josep Trenado Alvarez^309^, Vasiliki TRIANTAFYLLIA^310^, Sophie TROUILLET-ASSANT^311^, Jesús Troya^312^, Owen T Y Tsang^313^, Liina Tserel^314^, Eugene Y K Tso^315^, Alessandra Tucci^316^, Şadiye Kübra Tüter Öz^15^, Matilde Valeria Ursini^125^, Takanori Utsumi^225^, Yurdagul Uzunhan^317^, Pierre Vabres^318^, Juan Valencia-Ramos^319^, Ana Maria Van Den Rym^127^, Isabelle Vandernoot^320^, Valentina Velez-Santamaria^321^, Silvia Patricia Zuniga Veliz^134^, Mateus C Vidigal^322^, Sébastien Viel^253^, Cédric Vilain^323^, Marie E Vilaire-Meunier^223^, Judit Villar-García^324^, Audrey Vincent^57^, Guillaume Vogt^325^, Guillaume Voiriot^326^, Alla Volokha^327^, Fanny Vuotto^158^, Els Wauters^328^, Joost Wauters^329^, Alan K L Wu^330^, Tak-Chiu Wu^331^, Aysun Yahşi^332^, Osman YESILBAS^333^, Mehmet Yildiz^168^, Barnaby E Young^187^, Ufuk Yükselmiş^334^, Mayana Zatz^63^, Marco Zecca^39^, Valentina Zuccaro^62^, Van Praet Jens^335^, Lambrecht Bart N.^336^, Van Braeckel Eva^336^, Bosteels Cédric^336^, Hoste Levi^337^, Hoste Eric^338^, Fré Bauters^336^, Jozefien De Clercq^336^, Heijmans Cathérine^339^, Slabbynck Hans^340^, Naesens Leslie3^41^, Benoit Florkin^342^, Cécile Boulanger^343^, Dimitri Vanderlinden^344^

^1^Germans Trias i Pujol University Hospital and Research Institute, Badalona, Barcelona, Spain. ^2^Respiratory Diseases Division, IRCCS Policlinico San Matteo Foundation, University of Pavia, Pavia, Italy. ^3^Neonatal Intensive Care Unit, Fondazione IRCCS Policlinico San Matteo, Pavia, Italy. ^4^Navarra Health Service Hospital, Pamplona, Spain. ^5^Jeffrey Model Diagnostic and Research Center for Primary Immunodeficiencies, Barcelona, Catalonia, Spain, Immunology Division, Genetics Department, Vall d’Hebron University Hospital (HUVH), Vall d’Hebron Research Institute (VHIR), Vall d’Hebron Barcelona Hospital Campus, Universitat Autònoma de Barcelona (UAB), Barcelona, Catalonia, Spain. Catalonia, Barcelona, Spain. ^6^Immunohematology Unit, San Raffaele Hospital, Milan, Italy. ^7^Ondokuz Mayıs University Medical Faculty Pediatrics, Samsun, Turkey. ^8^Department of Infectious Diseases, Loghman Hakim Hospital, Shahid Beheshti University of Medical Sciences, Tehran, Iran. ^9^Hospital Regional de Huehuetenango, “Dr. Jorge Vides de Molina”, Guatemala. ^10^Hospital Nacional Edgardo Rebagliati Martins, Lima, Peru. ^11^Parc Sanitari Sant Joan de Déu, Sant Boi de Llobregat Spain. ^12^Khyber Medical University, Khyber Pakhtunkhwa, Pakistan. ^13^Anesthesia and Intensive Care, Rianimazione I, Fondazione IRCCS Policlinico San Matteo, Pavia, Italy. ^14^Virology Research Center, National institutes of Tuberculosis and Lung diseases, Shahid Beheshti University of Medical Sciences, Tehran, Iran. ^15^Department of Pediatrics, Division of Pediatric Infectious Diseases, Selcuk University Faculty of Medicine, Konya, Turkey. ^16^College of Medicine, Imam Abdulrahman Bin Faisal University, Dammam, Saudi Arabia; Department of Pediatrics, King Fahad Hospital of the University, Al-Khobar, Saudi Arabia. ^17^Intensive care unit, Hôpital Européen, Marseille, France. ^18^Immunology Department, Hospital 12 de Octubre, Research Institute imas12, Complutense University, Madrid, Spain. ^19^Immunology Department, Asturias Central University Hospital, Biosanitary Research Institute of the Principality of Asturias (ISPA), Oviedo, Spain. ^20^Emergency and Critical Care Medicine Departments, College of Medicine, Imam AbdulRahman Ben Faisal University, Dammam, Saudi Arabia. ^21^Clinical Immunology and Primary Immunodeficiencias Unit, Hospital Sant Joan de Déu, Institut de Recerca Sant Joan de Déu, Barcelona; Universitat de Barcelona, Barcelona, Spain. ^22^Department of Biological Immunology, Necker Hospital for Sick Children, APHP and INEM, Paris, France. ^23^Internal medicine department, Hôpital Lariboisière, APHP; Université de Paris, Paris, France. ^24^Internal medicine department, Pitié-Salpétrière Hospital, Paris, France. ^25^Department of Internal Medicine, Hospital Universitari de Bellvitge, IDIBELL, Barcelona, Spain. ^26^Service de Médecine Intensive Réanimation, Hôpitaux Universitaires Henri Mondor, AP-HP; Groupe de Recherche Clinique CARMAS, Faculté de Santé de Créteil, Université Paris Est Créteil, Créteil, France. ^27^INSERM U1163, University of Paris, Imagine Institute, Paris, France & Pediatric Neurology Department, Necker-Enfants malades Hospital, APHP, Paris, France. ^28^Hospital U. de Tarragona Joan XXIII. Universitat Rovira i Virgili (URV). IISPV, Tarragona, Spain. ^29^Department of Propedeutics of Pediatrics and Medical Genetics, Danylo Halytsky Lviv National Medical University, Lviv, Ukraine. ^30^Department of Immunology and Allergy, Konya City Hospital, Konya, Turkey. ^31^Private practice, Paris, France. ^32^INSERM U1109, University of Strasbourg, Strasbourg, France. ^33^Necker Hospital for Sick Children, AP-HP, Paris, France. ^34^Molecular Virology Unit, Microbiology and Virology Department, Fondazione IRCCS Policlinico San Matteo, Pavia, Italy. ^35^Department of Infectious Diseases, CHU de Caen, Caen, France. ^36^Consorcio Hospital General Universitario, Valencia, Spain. ^37^The Genetics Institute, Tel Aviv Sourasky Medical Center and Sackler Faculty of Medicine, Tel Aviv University, Tel Aviv, Israel. ^38^Dept Urology, Nephrology, Transplantation, APHP-SU, Sorbonne Université, INSERM U 1082, Paris, France. ^39^Cell Factory and Pediatric Hematology-Oncology, Fondazione IRCCS Policlinico San Matteo, Pavia, Italy. ^40^Yildirim Beyazit University, Faculty of Medicine, Ankara City Hospital, Children's Hospital, Ankara, Turkey. ^41^University of Lyon, CIRI, INSERM U1111, National referee center RAISE, Pediatric Rheumatology, HFME, Hospices Civils de Lyon, Lyon, France. ^42^Center for Clinical Immunology, CABA, Buenos Aires, Argentina. ^43^Cruces University Hospital, Bizkaia, Spain. ^44^Pediatric Immunology and Vaccinology Unit, Geneva University Hospitals and Faculty of Medicine, Geneva, Switzerland. ^45^University Hospital and Research Institute “Germans Trias i Pujol”, Badalona, Spain. ^46^Hematology, Georges Pompidou Hospital, APHP, Paris, France. ^47^Pediatric Infectious Diseases Unit, Instituto de Investigación Hospital 12 de Octubre (imas12), Hospital Universitario 12 de Octubre, Universidad Complutense, Madrid, Spain. ^48^Infectious disease Unit, Pitié-Salpêtrière Hospital, AP-AP, Paris, France. ^49^Department of Pediatrics, Thomayer’s Hospital, 1st Faculty of Medicine, Charles University, Prague, Czech Republic; Department of Immunology, Motol University Hospital, 2nd Faculty of Medicine, Charles University, Prague, Czech Republic. ^50^Centro de Investigación Biomédica en Red de Enfermedades Hepàticas y Digestivas (Ciberehd). Hospital de Mataró, Consorci Sanitari del Maresme, Mataró, Spain. ^51^Shupyk National Healthcare University of Ukraine, Kyiv, Ukraine. ^52^Service de Pneumologie, Hopital Bichat, APHP, Paris, France. ^53^Department of infectious diseases, CIC1408, GIMAP CIRI INSERM U1111, University Hospital of Saint-Etienne, Saint-Etienne, France. ^54^Clinical immunology unit, pediatric infectious disease departement, Faculty of Medicine and Pharmacy, Averroes University Hospital. LICIA Laboratoire d'immunologie clinique, d'inflammation et d'allergie, Hassann Ii University., Casablanca, Morocco. ^55^Bégin Military Hospital, St Mandé, France. ^56^Sorbonne Université, INSERM, Institut Pierre Louis d'Epidémiologie et de Santé Publique (iPLESP), AP-HP, Hôpital Pitié Salpêtrière, Service de Virologie, Paris, France. ^57^Endocrinology unit, APHP Hôpitaux Universitaires Paris-Sud, Le Kremlin-Bicêtre, France. ^58^Department of Children's Diseases and Pediatric Surgery, I.Horbachevsky Ternopil National Medical University, Ternopil, Ukraine. ^59^Pneumology Unit, Tenon Hospital, AP-HP, Paris, France. ^60^Department of Respiratory Diseases, Hospital Clínico y Universitario de Valencia, Valencia, Spain. ^61^Intensive care unit, Réseau Hospitalier Neuchâtelois, Neuchâtel, Switzerland. ^62^Infectious Diseases Unit, Fondazione IRCCS Policlinico San Matteo, Pavia, Italy. ^63^Human Genome and stem-cell research center- University of São Paulo, São Paulo, Brazil. ^64^Department of Internal Medicine, College of Medicine, Imam Abdulrahman Bin Faisal University, Dammam, Saudi Arabia. ^65^Hospital Insular, Las Palmas de Gran Canaria, Spain. ^66^MS Center, Spedali Civili, Brescia, Italy. ^67^Laboratoire d'ImmunoRhumatologie Moléculaire, plateforme GENOMAX, INSERM UMR_S 1109, Faculté de Médecine, ITI TRANSPLANTEX NG, Université de Strasbourg, Strasbourg, France. ^68^Fondazione IRCCS Ca' Granda Ospedale Maggiore Policlinico, Milan, Italy. ^69^Neuromuscular Unit. Neurology Department. Hospital Universitari de Bellvitge - IDIBELL and CIBERER, Barcelona, Spain. ^70^Hopital Robert Debré, Paris, France. ^71^Pediatric Immuno-hematology Unit, Necker Enfants Malades Hospital, AP-HP, Paris, France. ^72^Department of Infectious and Tropical Diseases, University of Brescia, ASST Spedali Civili di Brescia, Brescia, Italy. ^73^Doctoral Health Care Center, Canarian Health System, Las Palmas de Gran Canaria, Spain. ^74^Hôpital Foch, Suresnes, France. ^75^Selcuk University Faculty of Medicine, Department of Anesthesiology and Reanimation, Intensive Care Medicine Unit, Konya, Turkey. ^76^Division of Clinical Pharmacology and Toxicology, Institute of Pharmacological Sciences of Southern Switzerland, Ente Ospedaliero Cantonale & Faculty of Biomedical Sciences, Università della Svizzera italiana, Lugano, Switzerland. ^77^Necker Hospital for Sick Children, Paris University, AP-HP, Paris, France. ^78^Pasteur Institute, Paris, France. ^79^McGill University Health Centre, Montreal, Canada. ^80^University Hospital and Research Institute “Germans Trias i Pujol”, IrsiCaixa AIDS Research Institute, UVic-UCC, Badalona, Spain. ^81^Clinical Biochemistry, Pathology, Pediatric Neurology and Molecular Medicine Departments and Biobank, Institut de Recerca Sant Joan de Déu and CIBERER-ISCIII, Esplugues, Spain. ^82^AP-HP, Avicenne Hospital, Intensive Care Unit, Bobigny, France; University Sorbonne Paris Nord, Bobigny, France; INSERM, U942, F-75010, Paris, France. ^83^Hospital Universitari Vall d’Hebron, Barcelona, Spain. ^84^Pitié-Salpêtrière Hospital, Paris, France. ^85^Service de médecine Intensive Réanimation, Groupe Hospitalier Pitié-Salpêtrière, Sorbonne Université, France. ^86^Umraniye Training and Research Hospital, Istanbul, Turkey. ^87^Faculty of Medical Sciences at University “Goce Delcev”, Shtip, North Macedonia. ^88^Department of Biochemistry, College of Medicine, Imam Abdulrahman Bin Faisal University, Dammam, Saudi Arabia. ^89^Fundació Docencia i Recerca Mutua Terrassa, Barcelona, Spain. ^90^Maladies Infectieuses et Tropicales, Nouvel Hôpital Civil, CHU Strasbourg, Strasbourg, France. ^91^UNSW Medicine, St Vincent's Clinical School; Department of Thoracic Medicine, St Vincent's Hospital Darlinghurst, Sidney, Australia. ^92^Intensive Care unit, Montreuil hospital, Montreuil, France. ^93^CHU Saint-Pierre, Université Libre de Bruxelles (ULB), Brussels, Belgium. ^94^Pediatric Intensive Care Unit, Robert-Debré University Hospital, APHP, Paris, France. ^95^General Internal Medicine, University Hospitals Leuven, Leuven, Belgium. ^96^Hôpital Jean Verdier, APHP, Bondy, France. ^97^Specialized Immunology Laboratory of Dr. Shahrooei, Sina Medical Complex, Ahvaz, Iran. ^98^Centre de génétique humaine, CHU Besançon, Besançon, France. ^99^Sorbonne Université médecine and APHP Sorbonne université site Pitié-Salpêtrière, Paris, France. ^100^Pediatric Neurology Department, Necker-Enfants malades hospital, APHP, Paris, France. ^101^Department of Internal Medicine, Fondazione IRCCS Policlinico San Matteo, University of Pavia, Pavia, Italy. ^102^Intensive Care unit, Georges Pompidou Hospital, APHP, Paris, France. ^103^Department of Pneumology, AZ Delta, Roeselare, Belgium. ^104^Molecular Diagnostic Unit, Fundación Rioja Salud, Logroño, La Rioja, Spain. ^105^Bégin military Hospital, Saint Mandé, France. ^106^Department of Pediatrics, Institute of Clinical Sciences, The Sahlgrenska Academy, University of Gothenburg, Gothenburg, Sweden; Department of Rheumatology and Inflammation Research, Institute of Medicine, The Sahlgrenska Academy, University of Gothenburg, Gothenburg, Sweden. ^107^Bursa City Hospital, Bursa, Turkey. ^108^Department of Medical Biochemistry and Molecular Biology, Faculty of Medicine, Mansoura University, Mansoura, Egypt. ^109^Tokyo Medical and Dental University, Tokyo, Japan. ^110^Ondokuz Mayıs University Faculty of Medicine, Samsun, Turkey. ^111^Necmettin Erbakan University, Meram Medical Faculty, Division of Pediatric Allergy and Immunology, Konya, Turkey. ^112^University Donostia Hospital, Gipuzkoa, Spain. ^113^Internal Medicine, University Hospital Edouard Herriot, Hospices Civils de Lyon, Lyon, France. ^114^Centre de Génétique, CHU Dijon, Dijon, France. ^115^Robert Debré Hospital, Paris, France. ^116^APHP Tenon Hospital, Paris, France. ^117^Sorbonne Universités, UPMC University of Paris, Paris, France. ^118^Department of Clinical Immunology, Hospital Clínico San Carlos, Madrid, Spain. ^119^Intensive Care Department, Vall d’Hebron University Hospital (HUVH), Vall d’Hebron Barcelona Hospital Campus, Barcelona, Catalonia, Spain, Shock, Organ Dysfunction and Resuscitation Research Group. Vall d’Hebron Research Institute (VHIR), Vall d’Hebron Barcelona Hospital Campus, Barcelona, Catalonia, Spain. ^120^Intensive Care Unit, Hospital Clínico y Universitario de Valencia, Valencia, Spain. ^121^Genomics Division, Instituto Tecnológico y de Energías Renovables (ITER), Santa Cruz de Tenerife, Spain; CIBER de Enfermedades Respiratorias, Instituto de Salud Carlos III, Madrid, Spain; Research Unit, Hospital Universitario N.S. de Candelaria, Santa Cruz de Tenerife, Spain; Instituto de Tecnologías Biomédicas (ITB), Universidad de La Laguna, San Cristóbal de La Laguna, Spain, Santa Cruz de Tenerife, Spain. ^122^CHU Limoges and INSERM CIC 1435 & UMR 1092, Limoges, France. ^123^Infectious Diseases Unit, Department of Pediatrics, Hospital Sant Joan de Déu, Barcelona, Spain; Institut de Recerca Sant Joan de Déu, Spain; Universitat de Barcelona (UB), Barcelona, Spain. ^124^Department of Pathology, United Christian Hospital, Hong Kong. ^125^Institute of Genetics and Biophysics ‘Adriano Buzzati-Traverso’, IGB-CNR, Naples, Italy. ^126^Department of Pediatrics, Children's Hospital Zagreb, University of Zagreb School of Medicine, Zagreb, Josip Juraj Strossmayer University of Osijek, Medical Faculty Osijek, Osijek, Croatia. ^127^Laboratory of Immunogenetics of Human Diseases, IdiPAZ Institute for Health Research, La Paz Hospital, Madrid, Spain. ^128^Hematology, APHP, Hopital Européen Georges Pompidou and INSERM UMR-S1140, Paris, France. ^129^Faculty of Medicine, Department of Pediatrics, Division of Pediatric Infectious Diseases, Karadeniz Technical University, Trabzon, Turkey. ^130^Division of Immunology, Hospital General Universitario and Instituto de Investigación Sanitaria “Gregorio Marañón”, Madrid, Spain. ^131^Bégin military Hospital, Bégin, France. ^132^Aix Marseille Univ, IRD, AP-HM, SSA, VITROME, IHU Méditerranée Infection, Marseille, France, French Armed Forces Center for Epidemiology and Public Health (CESPA), Marseille, France. ^133^Pediatric Intensive Care Unit, Hospital Sant Joan de Déu, Barcelona, Spain. ^134^Guatemala. ^135^Department of Internal Medicine, Hôpital Erasme, Université Libre de Bruxelles, Brussels, Belgium. ^136^Immunodeficiencies Unit, Research Institute Hospital, madrid, Spain. ^137^Primary Immunodeficiencies Unit, Pediatrics, University Hospital 12 octubre, Madrid, Spain; School of Medicine Complutense University of Madrid, Madrid, Spain. ^138^Genomics Division, Instituto Tecnológico y de Energías Renovables (ITER), Santa Cruz de Tenerife, Spain. ^139^Assistance Publique Hôpitaux de Paris, Paris, France. ^140^Ankara City Hospital, Ankara, Turkey. ^141^Department of Intensive Care, Hospital Universitari de Bellvitge, IDIBELL, Barcelona, Spain. ^142^Immunodeficiency Outpatient Clinic, Institute for Medical Immunology, FOCIS Center of Excellence, Charité Universitätsmedizin Berlin, Germany. ^143^Surgical Intensive Care Unit, University Hospitals Leuven, Leuven, Belgium. ^144^CNAG-CRG, Barcelona Institute of Science and Technology, Barcelona, Spain. ^145^Department of Internal Medicine, National Reference Center for Rare Systemic Autoimmune Diseases, AP-HP, APHP-CUP, Hôpital Cochin, Paris, France. ^146^Department of Pediatric Immunology and Pulmonology, Center for Primary Immunodeficiency Ghent, Jeffrey Model Diagnosis and Research Center, PID research lab, Ghent University Hospital, Ghent, Belgium. ^147^Sharjah Institute of Medical Research, College of Medicine, University of Sharjah, Sharjah, UAE, Sharjah, UAE. ^148^Department of Biosciences and Nutrition, SE14183, Huddinge, Karolinska Institutet, Stockholm, Sweden. ^149^Department of Pediatrics (Infectious Diseases), Istanbul Faculty of Medicine, Istanbul University, Istanbul, Turkey. ^150^I. Horbachevsky Ternopil National Medical University, Ternopil, Ukraine. ^151^Pediatric Infectious Diseases Unit, Bakirkoy Dr. Sadi Konuk Training and Research Hospital, University of Health Sciences, Istanbul, Turkey. ^152^Health Sciences University, Darıca Farabi Education and Research Hospital, Kocaeli, Turkey. ^153^Department of Immunology, Hospital Universitario de Gran Canaria Dr. Negrín, Canarian Health System, Las Palmas de Gran Canaria, Spain. ^154^Department of Pediatrics, Queen Elizabeth Hospital, Hong Kong. ^155^IntensivenCare Unit. Marqués de Valdecilla Hospital, Santander, Spain. ^156^Hospital del Mar, Institut Hospital del Mar d'Investigacions Mèdiques (IMIM), UAB, UPF, Barcelona. ^157^Intensive care unit, APHM, Marseille, France. ^158^CHU Lille, Lille, France. ^159^Department of Medicine, The University of Hong Kong, Hong Kong. ^160^Department of Pediatrics, Columbia University, New York, NY, USA. ^161^Centre hospitalier intercommunal Poissy Saint Germain en Laye, Poissy, France. ^162^IHU Méditerranée Infection, Service de l'Information Médicale, Hôpital de la Timone, Marseille, France. ^163^Health Science University Ankara City Hospital, Ankara, Turkey. ^164^School of Medicine, General Surgery Department Fevzi Çakmak Mah, Marmara University, Istanbul, Turkey. ^165^Mersin City Education and Research Hospital, Mersin, Turkey. ^166^Division of Pediatric Infectious Diseases, Prof. Dr. Cemil Tascıoglu City Hospital, Istanbul, Turkey. ^167^Departments of Infectious Diseases and Clinical Microbiology, Bakirkoy Dr. Sadi Konuk Training and Research Hospital, University of Health Sciences, Istanbul, Turkey. ^168^Department of Pediatric Rheumatology, Istanbul University-Cerrahpasa, Istanbul, Turkey. ^169^Department of Pediatrics, Tokyo Medical and Dental University, Tokyo, Japan. ^170^Health Sciences University, Umraniye Education and Research Hospital, Istanbul, Turkey. ^171^Department of Parasitology and Research Center for Infectious Disease Sciences, Graduate School of Medicine, Osaka City University, Osaka, Japan. ^172^Pediatric Infectious Diseases Unit of Osman Gazi University Medical School in Eskişehir, Turkey. ^173^Meram Medical Faculty, Necmettin Erbakan University, Konya, Turkey. ^174^Department of Immunology, 2nd Faculty of Medicine, Charles University and University Hospital in Motol, Prague, Czech Republic. ^175^ICU, 1st Department of Respiratory Medicine, National and Kapodistrian University of Athens, Medical School, 'Sotiria' General Hospital of Chest Diseases, Athens, Greece. ^176^Central Clinical Hospital of the Ministry of Interior and Administration, Warsaw, Poland. ^177^Clinique des soins intensifs, HFR Fribourg, Fribourg, Switzerland. ^178^Oncobiologie Génétique Bioinformatique, PC Bio, CHU Besançon, Besançon, France. ^179^Department of Intensive Care, Tuen Mun Hospital, Hong Kong. ^180^Pediatric Infectious Disease Unit, Hospital Authority Infectious Disease Center, Princess Margaret Hospital, Hong Kong (Special Administrative Region), China. ^181^Department of Pathology, Queen Mary Hospital, Hong Kong. ^182^Aix Marseille Univ, IRD, MEPHI, IHU Méditerranée Infection, Marseille, France. ^183^Department of Pediatrics, Tuen Mun Hospital, Hong Kong. ^184^Biomedical Research Foundation of the Academy of Athens, Athens, Greece. ^185^Necker hospital, Paris, France. ^186^Department of Pediatrics and Adolescent Medicine, The University of Hong Kong, Hong Kong, China. ^187^National Centre for Infectious Diseases, Singapore. ^188^Hospital Universitario Reina Sofía, Cordoba, Spain. ^189^Imperial College, London, England. ^190^Hospital General San Juan de Dios, Ciudad de Guatemala, Guatemala. ^191^Endocrinology and diabetes for children, AP-HP, Bicêtre Paris-saclay hospital, Le Kremlin-Bicêtre, France. ^192^Innate Immunity group, IdiPAZ Institute for Health Research, La Paz Hospital, Madrid, Spain. ^193^Neurology unit, APHP Pitié-Salpêtrière Hospital, Paris University, Paris, France. ^194^Department of Medicine, Pamela Youde Nethersole Eastern Hospital, Hong Kong. ^195^Intensive care unit, APHP Pitié-Salpêtrière Hospital, Paris University, Paris, France. ^196^National Centre for Infectious Diseases; Tan Tock Seng Hospital; Yong Loo Lin School of Medicine; Lee Kong Chian School of Medicine, Singapore. ^197^Hospital de Niños Dr Ricardo Gutierrez, Buenos Aires, Argentina. ^198^Department of Clinical Immunology and Infectious Diseases, National Research Institute of Tuberculosis and Lung Diseases, Shahid Beheshti University of Medical Sciences,, Tehran, Iran. ^199^Neurooncology and Neuroinflammation Unit, IRCCS Mondino Foundation, Pavia, Italy. ^200^Clinical Tuberculosis and Epidemiology Research Center, National Research Institute of Tuberculosis and Lung Diseases (NRITLD), Shahid Beheshti University of Medical Sciences, Tehran, Iran. ^201^Coordenadora da Unidade de Infeciologia e Imunodeficiências do Serviço de Pediatria, Centro Materno-Infantil do Norte, Porto, Portugal. ^202^Hospital Sant Joan de Déu and University of Barcelona,, Barcelona, Spain. ^203^Pediatric Infectious Diseases and Immunodeficiencies Unit, Hospital Universitari Vall d’Hebron, Vall d’Hebron Research Institute, Vall d’Hebron Barcelona Hospital Campus, Universitat Autònoma de Barcelona (UAB), Barcelona, Catalonia, Spain. ^204^Hospital Universitari Mutua de Terrassa, Universitat de Barcelona, Barcelona, Spain. ^205^IrsiCaixa AIDS Research Institute, ICREA, UVic-UCC, Research Institute “Germans Trias i Pujol”, Badalona, Spain. ^206^Department of Laboratory, Cruces University Hospital, Barakaldo, Bizkaia, Spain, Bizkaia, Spain. ^207^Intensive Care Unit, Hospital General Universitario “Gregorio Marañón”, Madrid, Spain. ^208^University of New South Wales, Australia. ^209^APHP Pitié-Salpêtrière Hospital, Paris, France. ^210^Department of Pediatrics, Complejo Hospitalario Universitario Insular-Materno Infantil, Canarian Health System, Las Palmas de Gran Canaria, Spain. ^211^Medical Intensive Care Unit, University Hospitals Leuven, Leuven, Belgium. ^212^Aix-Marseille University, APHM, Marseille, France. ^213^Service de Médecine Intensive Réanimation, Hôpitaux Universitaires Henri Mondor, Assistance Publique - Hôpitaux de Paris (AP-HP). Groupe de Recherche Clinique CARMAS, Faculté de Santé de Créteil, Université Paris Est Créteil, France. ^214^APHP Cohin Hospital, Paris, France. ^215^Department of Critical Care Medicine, Ente Ospedaliero Cantonale, Bellinzona, Switzerland. ^216^Necmettin Erbakan University, Meram Medical Faculty, Division of Pediatric Infectious Diseases, Konya, Turkey. ^217^Department of Pediatrics, University Hospitals Leuven; KU Leuven, Department of Microbiology, Immunology and Transplantation; Laboratory for Inborn Errors of Immunity, KU Leuven, Leuven, Belgium. ^218^Hospices Civils de Lyon, Hôpital de la Croix-Rousse, Lyon, France. ^219^Hôpital Erasme, Brussels, Belgium. ^220^Centre hospitalier de gonesse, Gonesse, France. ^221^Aix Marseille Univ, IRD, AP-HM, MEPHI, IHU Méditerranée Infection, Marseille, France. ^222^Vascular Medicine, Georges Pompidou Hospital, APHP, Paris, France. ^223^Institut Jérôme Lejeune, Paris, France. ^224^Division of Pulmonary and Critical Care, College of Medicine-Jacksonville, University of Florida, Jacksonville, FL, USA. ^225^Department of Pediatrics, Hiroshima University Graduate School of Biomedical and Health Sciences, Hiroshima, Japan. ^226^BC Children's Hospital Research Institute, University of British Columbia, Vancouver, Canada. ^227^Médecine Intensive Réanimation, Hôpitaux Universitaires Henri Mondor, Assistance Publique – Hôpitaux de Paris (AP-HP), Créteil, France. ^228^Guanarteme Health Care Center, Canarian Health System, Las Palmas de Gran Canaria, Spain. ^229^Regional University Hospital of Malaga, Malaga, Spain. ^230^Department of Immunology, Hospital Universitari de Bellvitge, IDIBELL, Barcelona, Spain. ^231^Aix Marseille Univ, INSERM, INRAE, C2VN, Marseille, France. ^232^Department of General Pediatrics, Hôpital Bicêtre, AP-HP, University of Paris Saclay, Le Kremlin-Bicêtre, France. ^233^INSERM U1144, Université de Paris, DMU INVICTUS, APHP.Nord, Département de Médecine Interne, Lariboisière Hospital, Paris, France. ^234^CHU de La Timone, Marseille, France. ^235^Department of Epidemiology, Infectious Disease Control and Prevention, Graduate School of Biomedical and Health Sciences, Hiroshima University, Hiroshima, Japan. ^236^Pediatric Immunology and rhumatology Department,Necker Hospital, AP-HP, Paris, France. ^237^Centro Hospitalar Universitário de Lisboa Central, Lisbon, Portugal. ^238^Infectious Diseases Horizontal Technlogy Centre, A*STAR; Singapore Immunology Network, A*STAR, Singapore. ^239^Department of Medicine and Geriatrics, Tuen Mun Hospital, Hong Kong. ^240^Regional Universitary Hospital of Malaga, Málaga, Spain. ^241^Department of Immunology, Hospital Universitario Marqués de Valdecilla, Santander, Spain. ^242^Bilkent University, Department of Molecular Biology and Genetics, Ankara, Turkey. ^243^BRFAA, Athens, Greece. ^244^IHU Méditerranée Infection, Aix Marseille Univ, IRD, AP-HM, SSA, VITROME, IHU Méditerranée Infection, Marseille, France. ^245^L'Hôpital Foch, Suresnes, France. ^246^Department of Immunology, CIC1408, GIMAP CIRI INSERM U1111, University Hospital of Saint-Etienne, St Etienne, France. ^247^Department of Immunology, Hospital Universitario 12 de Octubre, Instituto de Investigación Sanitaria Hospital 12 de Octubre (imas12), Madrid, Spain. ^248^Mexico. ^249^Diabetes Research Institute, IRCCS San Raffaele Hospital, Milan, Italy. ^250^APHP Hôpitaux Universitaires Paris-Sud, Le Kremlin-Bicêtre, France. ^251^AP-HP, Avicenne Hospital, Intensive Care Unit, Bobigny, France; INSERM UMR-S 942, Cardiovascular Markers in Stress Conditions (MASCOT), University of Paris, Paris, France. ^252^Neurometabolic Diseases Laboratory, IDIBELL-Hospital Duran i Reynals, Barcelona; CIBERER U759, ISCiii Madrid, Spain. ^253^Hospices Civils de Lyon, Lyon, France. ^254^Univ. Lille, INSERM U1285, CHU Lille, Pôle de médecine intensive-réanimation, CNRS, UMR 8576 - Unité de Glycobiologie Structurale et Fonctionnelle, Lille, France. ^255^Department of General pediatrics, Robert Debre Hospital, Paris, France. ^256^University of British Columbia, Vancouver, Canada. ^257^Jeffrey Model Diagnostic and Research Center for Primary Immunodeficiencies, Barcelona, Catalonia, Spain, Diagnostic Immunology Research Group, Vall d’Hebron Research Institute (VHIR), Vall d’Hebron University Hospital (HUVH), Vall d’Hebron Barcelona Hospital Campus, Barcelona, Catalonia, Spain. ^258^AP-HP, Avicenne Hospital, Intensive Care Unit, Bobigny, France; University Sorbonne Paris Nord, Bobigny, France. ^259^Centre Hospitalier de Saint-Denis, St Denis, France. ^260^Precision Medicine Unit, Lausanne University Hospital and University of Lausanne, Lausanne, Switzerland. ^261^Paris Cardiovascular Center, PARCC, INSERM, Université de Paris, Paris, France. ^262^Germans Trias i Pujol Hospital, Badalona, Spain. ^263^Medical intensive care unit. Hopital de la Croix-Rousse. Hospices Civils de Lyon, Lyon, France. ^264^Department of Clinical Laboratory, Hospital Universitari de Bellvitge, IDIBELL, Barcelona, Spain. ^265^Pediatric Infectious Diseases and Immunodeficiencies Unit, Hospital Universitari Vall d’Hebron, Vall d'Hebron Research Institute, Vall d’Hebron Barcelona Hospital Campus., Barcelona, Spain. ^266^Université de Paris, CNRS UMR-8601; Team Chemistry & Biology, Modeling & Immunology for Therapy, CBMIT, Paris, France. ^267^Germans Trias i Pujol University Hospital and Research Institute. Badalona, Badalona, Spain. ^268^Department of Immunology, University Hospital of Gran Canaria Dr. Negrín, Canarian Health System, Las Palmas de Gran Canaria, Spain; Department of Clinical Sciences, University Fernando Pessoa Canarias, Las Palmas de Gran Canaria, Spain. ^269^Neurometabolic Diseases Laboratory, Bellvitge Biomedical Research Institute (IDIBELL), 08908 L'Hospitalet de Llobregat; University Hospital Germans Trias i Pujol, Badalona, Barcelona, Catalonia, Spain. ^270^Consorcio Hospital General Universitario, Valencia, Spain. ^271^APHP Hôpitaux Universitaires Paris-Sud, Paris, France. ^272^Intensive Care Unit, Louis-Mourier Hospital, Colombes, France. ^273^Virology unit, Université de Paris, Cohin Hospital, APHP, Paris, France. ^274^Neurometabolic Diseases Laboratory and CIBERER U759, Barcelona, Spain. ^275^Hospital San Pedro, Logroño, Spain. ^276^University of Tartu, Institute of Biomedicine and Translational Medicine, Tartu, Estonia. ^277^Respiratory medicine, Georges Pompidou Hospital, APHP, Paris, France. ^278^Infectious Diseases Department, International Health Program of the Catalan Insitute of Health (PROSICS), Vall d’Hebron University Hospital (HUVH), Vall d’Hebron Barcelona Hospital Campus, Universitat Autónoma de Barcelona, Barcelona, Spain. ^279^Hospital Clínico San Carlos and IdSSC, Madrid, Spain. ^280^Faculdades Pequeno Príncipe, Instituto de Pesquisa Pelé Pequeno Príncipe, Curitiba, Brazil. ^281^AP-HP, Avicenne Hospital, Intensive Care Unit, Bobigny, France. ^282^Service de Médecine Intensive Réanimation, Institut de Cardiologie, Hopital Pitié-Salpêtrière, Paris, France. ^283^Department of Pediatrics, Medizinische Fakultät Carl Gustav Carus, Technische Universität Dresden, Dresden, Germany. ^284^CHRU de Nancy, Hôpital d'Enfants, Vandoeuvre, France. ^285^Chair of Nephrology, University of Brescia, Brescia, Italy. ^286^Department of Immunology, 2nd Faculty of Medicine, Charles University and Motol University Hospital, Prague, Czech Republic. ^287^Clínica Universidad de Navarra and Ciberes, Madrid, Spain. ^288^HUS Helsinki University Hospital, Children and Adolescents, Rare Disease Center, and Inflammation Center, Adult Immunodeficiency Unit, Majakka, Helsinki, Finland. ^289^Fundació Docencia i Recerca Mutua Terrassa, Terrassa, Spain. ^290^D.Rogachev National Medical and Research Center of Pediatric Hematology, Oncology, Immunoogy, Moscow, Russia. ^291^Haematology Laboratory, Lariboisière Hospital, University of Paris, Paris, France. ^292^Biomedical Research Foundation of the Academy of Athens. ^293^INSERM U1140, University of Paris, European Georges Pompidou Hospital, Paris, France. ^294^Department of Pediatrics, Faculty of Medicine, Mansoura University, Mansoura, Egypt. ^295^Critical Care Unit, Hospital Universitario de Gran Canaria Dr. Negrín, Canarian Health System, Las Palmas de Gran Canaria, Spain. ^296^CHU de Saint Etienne, Saint-Priest-en-Jarez, France. ^297^Pediatric Infectious Diseases and Immunodeficiencies Unit, Hospital Universitari Vall d’Hebron, Vall d'Hebron Research Institute, Vall d’Hebron Barcelona Hospital Campus. Universitat Autònoma de Barcelona (UAB). Barcelona, Catalonia, Spain, EU., Barcelona, Spain. ^298^Department of pediatric infectious diseases and pediatric immunology, Shupyk National Healthcare University of Ukraine, Kyiv, Ukraine. ^299^Gustave Roussy Cancer Campus, Villejuif, France. ^300^Intensive Care Unit, Avicenne Hospital, APHP, Bobigny, France. ^301^Laboratory of Immunology and Histocompatibility, Saint-Louis Hospital, Paris University, Paris, France. ^302^Center for Inflammation Research, Laboratory of Molecular Signal Transduction in Inflammation, VIB, Ghent, Belgium. ^303^Department of Internal Medicine, Université de Paris, INSERM, U970, PARCC, F-75015, Paris, France. ^304^Service de médecine intensive réanimation, CHU de Saint-Etienne, France. ^305^Dept of Rheumatology and Inflammation Research, Institute of Medicine, Sahlgrenska Academy, University of Gothenburg, Gothenburg, Sweden. ^306^University of Management and Technology, Lahore, Pakistan. ^307^Department of Infectious Diseases, Aarhus University Hospital, Aarhus, Denmark. ^308^First Division of Anesthesiology and Critical Care Medicine, University of Brescia, ASST Spedali Civili di Brescia, Brescia, Italy. ^309^Intensive Care Department, Hospital Universitari MutuaTerrassa, Universitat Barcelona, Terrassa, Spain. ^310^Laboratory of Immunobiology, Center for Clinical, Experimental Surgery and Translational Research, Biomedical Research Foundation of the Academy of Athens, Athens, Greece. ^311^International Center of Research in Infectiology, Lyon University, INSERM U1111, CNRS UMR 5308, ENS, UCBL, Lyon, France; Hospices Civils de Lyon, Lyon Sud Hospital, Pierre-Bénite, France. ^312^Infanta Leonor University Hospital, Madrid, Spain. ^313^Department of Medicine and Geriatrics, Princess Margaret Hospital, Hong Kong. ^314^University of Tartu, Institute of Clinical Medicine, Tartu, Estonia. ^315^Department of Medicine, United Christian Hospital, Hong Kong. ^316^Hematology Department, ASST Spedali Civili di Brescia, Brescia, Italy. ^317^Pneumologie, Hôpital Avicenne, APHP, INSERM U1272, Université Sorbonne Paris Nord, Bobigny, France. ^318^Dermatology unit, Laboratoire GAD, INSERM UMR1231 LNC, université de Bourgogne, Dijon, France. ^319^University Hospital of Burgos, Burgos, Spain. ^320^Center of Human Genetics, Hôpital Erasme, Université Libre de Bruxelles, Brussels, Belgium. ^321^Bellvitge University Hospital, L'Hospitalet de Llobregat, Barcelona, Spain. ^322^University of São Paulo, São Paulo, Brazil. ^323^CHU de Caen, Caen, France. ^324^Hospital del Mar - IMIM Biomedical Research Institute, Barcelona, Catalonia, Spain. ^325^Neglected Human Genetics Laboratory, INSERM, University of Paris, Paris, France. ^326^Sorbonne Université, Service de Médecine Intensive Réanimation, Hôpital Tenon, Assistance Publique-Hôpitaux de Paris, Paris, France. ^327^Pediatric Infectious Disease and Pediatric Immunology Department, Shupyk National Healthcare University of Ukraine, Kyiv, Ukraine. ^328^Department of Pneumology, University Hospitals Leuven, Leuven, Belgium. ^329^Laboratory for Clinical Infectious and Inflammatory Disorders, Departement of Microbiology, Immunology and Transplantation, Leuven, Belgium. ^330^Department of Clinical Pathology, Pamela Youde Nethersole Eastern Hospital, Hong Kong. ^331^Department of Medicine, Queen Elizabeth Hospital, Hong Kong. ^332^Ankara City Hospital, Children's Hospital, Ankara, Turkey. ^333^Division of Pediatric Infectious Disease, Department of Pediatrics, Faculty of Medicine, Karadeniz Technical University, Trabzon, Turkey. ^334^Health Sciences University, Lütfi Kırdar Kartal Education and Research Hospital, İstanbul, Turkey. ^335^Department of Nephrology and Infectiology, AZ Sint-Jan, Bruges, Belgium. ^336^Department of Pulmonology, Ghent University Hospital, Belgium. ^337^Department of Pediatric pulmonology and immunology, Ghent University Hospital, Belgium. ^338^Department of Intensive Care Unit, Ghent University Hospital, Belgium. ^339^Department of Pediatric hemato-oncology, Jolimont Hospital; Department of Pediatric hemato-oncology, HUDERF, Brussels, Belgium. ^340^Department of Pulmonology, ZNA Middelheim, Antwerp, Belgium. ^341^Department of Internal Medicine, Ghent University Hospital, Belgium. ^342^Department of Pediatric immuno-hémato-rhumatology, CHR Citadelle, Liége, Belgium. ^343^Department of Pediatric hemato-oncology, UCL Louvain, Belgium. ^344^Department of Pediatrics, Saint Luc, UCL Louvain, Belgium.

## Imagine COVID Group

Jean-Philippe Annereau^1^, Luis Briseño-Roa^1^, Olivier Gribouval^2^, Anna Pelet^2^

^1^Medetia Pharmaceuticals, Paris, France. ^2^Imagine Institute, Université de Paris, INSERM UMR 1163, Paris, France.

## French COVID Cohort Study Group

Laurent ABEL^1^, Claire ANDREJAK^2^, François ANGOULVANT^3^, Delphine BACHELET^4^, Marie BARTOLI^5^, Romain BASMACI^6^, Sylvie BEHILILL^7^, Marine BELUZE^8^, Dehbia BENKERROU^9^, Krishna BHAVSAR^4^, Lila BOUADMA^4^, Sabelline BOUCHEZ^10^, Maude BOUSCAMBERT^11^, Minerva CERVANTES-GONZALEZ^4^, Anissa CHAIR^4^, Catherine CHIROUZE^12^, Alexandra COELHO^13^, Camille COUFFIGNAL^4^, Sandrine COUFFIN-CADIERGUES^14^, Eric d’ORTENZIO^5^, Marie-Pierre DEBRAY^4^, Lauren DECONINCK^4^, Dominique DEPLANQUE^15^, Diane DESCAMPS^4^, Mathilde DESVALLÉE^16^, Alpha DIALLO^5^, Alphonsine DIOUF^13^, Céline DORIVAL^9^, François DUBOS^17^, Xavier DUVAL^4^, Brigitte ELHARRAR^18^, Philippine ELOY^4^, Vincent ENOUF^7^, Hélène ESPEROU^14^, Marina ESPOSITO-FARESE^4^, Manuel ETIENNE^19^, Eglantine FERRAND DEVOUGE^19^, Nathalie GAULT^4^, Alexandre GAYMARD^11^, Jade GHOSN^4^, Tristan GIGANTE^20^, Morgane GILG^20^, Jérémie GUEDJ^21^, Alexandre HOCTIN^13^, Isabelle HOFFMANN^4^, Ikram HOUAS^14^, Jean-Sébastien HULOT^22^, Salma JAAFOURA^14^, Ouifiya KAFIF^4^, Florentia KAGUELIDOU^23^, Sabrina KALI^4^, Antoine KHALIL^4^, Coralie KHAN^16^, Cédric LAOUÉNAN^4^, Samira LARIBI^4^, Minh LE^4^, Quentin LE HINGRAT^4^, Soizic LE MESTRE^5^, Hervé LE NAGARD^24^, François-Xavier LESCURE^4^, Sophie LETROU^4^, Yves LEVY^25^, Bruno LINA^11^, Guillaume LINGAS^24^, Jean Christophe LUCET^4^, Denis MALVY^26^, Marina MAMBERT^13^, France MENTRÉ^4^, Amina MEZIANE^9^, Hugo MOUQUET^7^, Jimmy Mullaert^4^, Nadège NEANT^24^, Duc NGUYEN^26^, Marion NORET^27^, Saad NSEIR^17^, Aurélie PAPADOPOULOS^14^, Christelle PAUL^5^, Nathan PEIFFER-SMADJA^4^, Thomas PERPOINT^28^, Ventzislava PETROV-SANCHEZ^5^, Gilles PEYTAVIN^4^, Huong PHAM^4^, Olivier PICONE^6^, Valentine PIQUARD^4^, Oriane PUÉCHAL^29^, Christian RABAUD^30^, Manuel ROSA-CALATRAVA^11^, Bénédicte ROSSIGNOL^20^, Patrick ROSSIGNOL^30^, Carine ROY^4^, Marion SCHNEIDER^4^, Richa SU^4^, Coralie TARDIVON^4^, Marie-Capucine TELLIER^4^, François TÉOULÉ^9^, Olivier TERRIER^11^, Jean-François TIMSIT^4^, Christelle TUAL^31^, Sarah TUBIANA^4^, Sylvie VAN DER WERF^7^, Noémie VANEL^32^, Aurélie VEISLINGER^31^, Benoit VISSEAUX^4^, Aurélie WIEDEMANN^25^, Yazdan YAZDANPANAH^4^

^1^INSERM UMR 1163, Paris, France. ^2^CHU Amiens, France. ^3^Hôpital Necker, Paris, France. ^4^Hôpital Bichat, Paris, France. ^5^ANRS, Paris, France. ^6^Hôpital Louis Mourier, Colombes, France. ^7^Pasteur Institute, Paris, France. ^8^F-CRIN Partners Platform, Paris, France. ^9^INSERM UMR 1136, Paris, France. ^10^CHU Nantes, France. ^11^INSERM UMR 1111, Lyon, France. ^12^CHRU Jean Minjoz, Besançon, France. ^13^INSERM UMR 1018, Paris, France. ^14^INSERM sponsor, Paris, France. ^15^Centre d'Investigation Clinique, INSERM CIC 1403, Centre Hospitalo universitaire de Lille, Lille, France. ^16^INSERM UMR 1219, Bordeaux, France. ^17^CHU Lille, France. ^18^CHI de Créteil, France. ^19^CHU Rouen, France. ^20^F-CRIN INI-CRCT, Nancy, France. ^21^Université de Paris, INSERM, IAME, F-75018 Paris, France. ^22^Hôpital Européen Georges Pompidou, Paris, France. ^23^Hôpital Robert Debré, Paris, France. ^24^INSERM UMR 1137, Paris, France. ^25^Vaccine Research Insitute (VRI), INSERM UMR 955, Créteil, France. ^26^CHU Bordeaux, France. ^27^RENARCI, Annecy, France. ^28^CHU Lyon, France. ^29^REACTing, Paris, France. ^30^CHU Nancy, France. ^31^INSERM CIC-1414, Rennes, France. ^32^Hôpital la Timone, Marseille, France.

## CoV-Contact Cohort

Loubna Alavoine^1^, Sylvie Behillil^2^, Charles Burdet^3^, Charlotte Charpentier^4^, Aline Dechanet^5^, Diane Descamps^6^, Xavier Duval^7^, Jean-Luc Ecobichon^1^, Vincent Enouf^8^, Wahiba Frezouls^1^, Nadhira Houhou^5^, Ouifiya Kafif^5^, Jonathan Lehacaut^1^, Sophie Letrou^1^, Bruno Lina^9^, Jean-Christophe Lucet^10^, Pauline Manchon^5^, Mariama Nouroudine^1^, Valentine Piquard^5^, Caroline Quintin^1^, Michael Thy^11^, Sarah Tubiana^1^, Sylvie van der Werf^8^, Valérie Vignali^1^, Benoit Visseaux^10^, Yazdan Yazdanpanah^10^, Abir CHAHINE^12^, Nawal WAUCQUIER^12^, Maria-Claire MIGAUD^12^, Dominique DEPLANQUE^12^, Félix DJOSSOU^13^, Mayka Mergeay-Fabre^14^, Aude LUCARELLI^15^, Magalie DEMAR^13^, Léa Bruneau^16^, Patrick Gérardin^17^, Adrien Maillot^16^, Christine Payet^18^, Bruno Laviolle^19^, Fabrice Laine^19^, Christophe Paris^19^, Mireille Desille-Dugast^19^, Julie Fouchard^19^, Denis MALVY^20^, Duc NGUYEN^20^, Thierry PISTONE^20^, Pauline PERREAU^20^, Valérie GISSOT^21^, Carole LE GOAS^21^, Samatha Montagne^22^, Lucie Richard^23^, Catherine Chirouze^24^, Kévin Bouiller^24^, Maxime Desmarets^25^, Alexandre Meunier^26^, Benjamin Lefévre^27^, Hélène Jeulin^28^, Karine Legrand^29^, Sandra Lomazzi^30^, Bernard Tardy^31^, Amandine Gagneux-Brunon^32^, Frédérique Bertholon^33^, Elisabeth Botelho-Nevers^32^, KOUAKAM Christelle KOUAKAM Christelle^34^, LETURQUE Nicolas LETURQUE Nicolas^34^, Layidé Roufai^34^, Karine Amat^35^, Sandrine Couffin-Cadiergues^34^, Hélène Espérou^36^, Samia Hendou^34^

^1^Centre d'Investigation Clinique, INSERM CIC 1425, Hôpital Bichat Claude Bernard, APHP, Paris, France. ^2^Institut Pasteur, Paris, France. ^3^Université de Paris, IAME, INSERM U1137, Paris, France, Hôpital Bichat Claude Bernard, APHP, Paris, France. ^4^Service de Virologie, Université de Paris, INSERM, IAME, UMR 1137, Hôpital Bichat Claude Bernard, APHP, Paris, France. ^5^Hôpital Bichat Claude Bernard, APHP, Paris, France. ^6^IAME INSERM U1140, Hôpital Bichat Claude Bernard, APHP, Paris, France. ^7^Centre d'Investigation Clinique, INSERM CIC 1425, APHP, IAME, Paris University, Paris, France. ^8^Institut Pasteur, U3569 CNRS, Université de Paris, Paris, France. ^9^Virpath Laboratory, International Center of Research in Infectiology, Lyon University, INSERM U1111, CNRS U5308, ENS, UCBL, Lyon, France. ^10^IAME INSERM U1138, Hôpital Bichat Claude Bernard, APHP, Paris, France. ^11^Center for Clinical Investigation, Assistance Publique-Hôpitaux de Paris, Bichat-Claude Bernard University Hospital, Paris, France. ^12^Centre d'Investigation Clinique, INSERM CIC 1403, Centre Hospitalo universitaire de Lille, Lille, France. ^13^Service des maladies infectieuses, Centre Hospitalo universitaire de Cayenne, Guyane, France. ^14^Centre d'Investigation Clinique, INSERM CIC 1424, Centre Hospitalier de Cayenne, Cayenne, Guyane Française. ^15^Service Hôpital de jour Adulte, Centre Hospitalier de Cayenne, Guyane, France. ^16^Centre d'Investigation Clinique, INSERM CIC 1410, Centre Hospitalo universitaire de la Réunion, La Réunion, France. ^17^Centre d'Investigation Clinique, INSERM CIC 1410, CHU Reunion, Saint-Pierre, Reunion island. ^18^Centre d'Investigation Clinique, INSERM CIC 1410, Centre de Ressources Biologiques, Centre Hospitalo universitaire de la Réunion, La Réunion, France. ^19^Centre d'Investigation Clinique, INSERM CIC 1414, Centre Hospitalo universitaire de Rennes, Rennes, France. ^20^Service des maladies infectieuses, Centre Hospitalo universitaire de Bordeaux, Bordeaux, France. ^21^Centre d'Investigation Clinique, INSERM CIC 1415, CHRU Tours, Tours, France. ^22^CRBT, Centre Hospitalo universitaire de Tours, Tours, France. ^23^Pole de Biologie Médicale, Centre Hospitalo universitaire de Tours, Tours, France. ^24^Service des maladies infectieuses, Centre Hospitalo universitaire de Besançon, Besançon, France. ^25^Service des maladies infectieuses, Centre d'investigation clinique, INSERM CIC1431, Centre Hospitalier Universitaire de Besançon, Besançon, France. ^26^Centre de Ressources Biologiques - Filière Microbiologique de Besançon, Centre Hospitalier Universitaire, Besançon, France. ^27^Université de Lorraine, CHRU-Nancy and APEMAC, Infectious and tropical diseases, Nancy, France. ^28^Laboratoire de Virologie, CHRU de Nancy Brabois, Vandoeuvre-lès-Nancy, France. ^29^INSERM CIC-EC 1433, Centre Hospitalo universitaire de Nancy, Nancy, France. ^30^Centre de ressources Biologiques, Centre Hospitalo universitaire de Nancy, Nancy, France. ^31^Centre d'Investigation Clinique, INSERM CIC 1408, Centre Hospitalo universitaire de Saint Etienne, Saint Etienne, France. ^32^Service des maladies infectieuses, Centre Hospitalo universitaire de Saint Etienne, Saint Etienne, France. ^33^Service des maladies infectieuses, CRB^42^-BTK, Centre Hospitalo Universitaire de Saint Etienne, Saint Etienne, France. ^34^Pole Recherche Clinique, INSERM, Paris France. ^35^IMEA Fondation Léon M'Ba, Paris, France. ^36^INSERM Clinical research Department, Paris, France.

## Amsterdam UMC Covid-19 Biobank

Michiel van Agtmael^2^, Anne Geke Algera^1^, Brent Appelman^2^, Frank van Baarle^1^, Diane Bax^3^, Martijn Beudel^4^, Harm Jan Bogaard^5^, Marije Bomers^2^, Peter Bonta^5^, Lieuwe Bos^1^, Michela Botta^1^, Justin de Brabander^2^, Godelieve de Bree^2^, Sanne de Bruin^1^, David T.P. Buis^1^, Marianna Bugiani^5^, Esther Bulle^1^, Osoul Chouchane^2^ Alex Cloherty^3^, Mirjam Dijkstra^12^, Dave A. Dongelmans^1^, Romein W.G. Dujardin^1^, Paul Elbers^1^, Lucas Fleuren^1^, Suzanne Geerlings^2^ Theo Geijtenbeek^3^, Armand Girbes^1^, Bram Goorhuis^2^, Martin P. Grobusch^2^, Florianne Hafkamp^3^, Laura Hagens^1^, Jorg Hamann^7^, Vanessa Harris^2^, Robert Hemke^8^, Sabine M. Hermans^2^ Leo Heunks^1^, Markus Hollmann^6^, Janneke Horn^1^, Joppe W. Hovius^2^, Menno D. de Jong^9^, Rutger Koning^4^, Endry H.T. Lim^1^, Niels van Mourik^1^, Jeaninne Nellen^2^, Esther J. Nossent^5^, Frederique Paulus^1^, Edgar Peters^2^, Dan A.I. Pina-Fuentes^4^, Tom van der Poll^2^, Bennedikt Preckel^6^, Jan M. Prins^2^, Jorinde Raasveld^1^, Tom Reijnders^2^, Maurits C.F.J. de Rotte^12^, Michiel Schinkel^2^, Marcus J. Schultz^1^, Femke A.P. Schrauwen^12^, Alex Schuurmans^10^, Jaap Schuurmans^1^, Kim Sigaloff^1^, Marleen A. Slim^1,2^, Patrick Smeele^5^, Marry Smit^1^, Cornelis S. Stijnis^2^, Willemke Stilma^1^, Charlotte Teunissen^11^, Patrick Thoral^1^, Anissa M Tsonas^1^, Pieter R. Tuinman^2^, Marc van der Valk^2^, Denise Veelo^6^, Carolien Volleman^1^, Heder de Vries^1^, Lonneke A. Vught^1,2^, Michèle van Vugt^2^, Dorien Wouters^12^, A. H (Koos) Zwinderman^13^, Matthijs C. Brouwer^4^, W. Joost Wiersinga^2^, Alexander P.J. Vlaar^1^, Diederik van de Beek (d.vandebeek@amsterdamumc.nl)^4^.

^1^Department of Intensive Care, Amsterdam UMC, Amsterdam, The Netherlands; ^2^Department of Infectious Diseases, Amsterdam UMC, Amsterdam, The Netherlands; ^3^Experimental Immunology, Amsterdam UMC, Amsterdam, The Netherlands; ^4^Department of Neurology, Amsterdam UMC, Amsterdam Neuroscience, Amsterdam, The Netherlands; ^5^Department of Pulmonology, Amsterdam UMC, Amsterdam, The Netherlands; ^6^Department of Anesthesiology, Amsterdam UMC, Amsterdam, The Netherlands; ^7^Amsterdam UMC Biobank Core Facility, Amsterdam UMC, Amsterdam, The Netherlands; ^8^Department of Radiology, Amsterdam UMC, Amsterdam, The Netherlands; ^9^Department of Medical Microbiology, Amsterdam UMC, Amsterdam, The Netherlands; ^10^Department of Internal Medicine, Amsterdam UMC, Amsterdam, The Netherlands; ^11^Neurochemical Laboratory, Amsterdam UMC, Amsterdam, The Netherlands; ^12^Department of Clinical Chemistry, Amsterdam UMC, Amsterdam, The Netherlands; ^13^Department of Clinical Epidemiology, Biostatistics and Bioinformatics, Amsterdam UMC, Amsterdam, The Netherlands.

## NIAID-USUHS COVID Study Group

Miranda F. Tompkins^1^, Camille Alba^1^, Andrew L. Snow^2^, Daniel N. Hupalo^1^, John Rosenberger^1^, Gauthaman Sukumar^1^, Matthew D. Wilkerson^1^, Xijun Zhang^1^, Justin Lack^3^, Andrew J. Oler^4^, Kerry Dobbs^5^, Ottavia M. Delmonte^5^, Jeffrey J. Danielson^5^, Andrea Biondi^6^, Laura Rachele Bettini^6^, Mariella D’Angio’^6^, Ilaria Beretta^7^, Luisa Imberti^8^, Alessandra Sottini^8^, Virginia Quaresima^8^, Eugenia Quiros-Roldan^9^, Camillo Rossi^10^

^1^The American Genome Center, Uniformed Services University of the Health Sciences; Henry M. Jackson Foundation for the Advancement of Military Medicine, Bethesda, USA. ^2^Department of Pharmacology & Molecular Therapeutics, Uniformed Services University of the Health Sciences, Bethesda, USA. ^3^NIAID Collaborative Bioinformatics Resource, Frederick National Laboratory for Cancer Research, Leidos Biomedical Research, Inc. Frederick, USA. ^4^Bioinformatics and Computational Biosciences Branch, Office of Cyber Infrastructure and Computational Biology, NIAID, NIH, Bethesda, USA. ^5^Laboratory of Clinical Immunology and Microbiology, Division of Intramural Research, NIAID, NIH, Bethesda, USA. ^6^Pediatric Departement and Centro Tettamanti-European Reference Network PaedCan, EuroBloodNet, MetabERN-University of Milano-Bicocca-Fondazione MBBM-Ospedale, San Gerardo, Monza, Italy. ^7^Department of Infectious Diseases, University of Milano-Bicocca, San Gerardo Hospital, Monza, Italy. ^8^CREA Laboratory, Diagnostic Department, ASST Spedali Civili di Brescia, Brescia, Italy. ^9^Department of Infectious and Tropical Diseases, University of Brescia and ASST Spedali Civili di Brescia, Brescia, Italy. ^10^Chief Medical Officer, ASST Spedali Civili di Brescia, Brescia, Italy.
